# Secrecy Performance Enhancement Using Self-Interference Cancellation in Wireless Mutual Broadcast Networks for Proximity-Based Services

**DOI:** 10.3390/s24113389

**Published:** 2024-05-24

**Authors:** Taesoo Kwon, HyeonWoo LEE

**Affiliations:** 1 Department of Computer Science and Engineering, Seoul National University of Science and Technology (SeoulTech), Seoul 01811, Republic of Korea; 2 Department of Mobile Systems Engineering, Dankook University, Yongin 16890, Republic of Korea; woojaa@dankook.ac.kr

**Keywords:** proximity-based services, neighbor discovery, physical layer security, 6G IoT, self-interference cancellation, full duplex, wireless broadcast, stochastic geometry, random access

## Abstract

With the increasing demand for data exchange between nearby devices in proximity-based services, enhancing the security of wireless mutual broadcast (WMB) networks is crucial. However, WMB networks are inherently vulnerable to eavesdropping due to the open broadcast nature of their communication. This paper investigates the improvement of secrecy performance in random-access-based WMB (RA-WMB) networks by integrating physical layer security (PLS) techniques with hybrid duplex (HBD) operations under a stochastic geometry framework. The HBD method balances half-duplex (HD) receiving and full-duplex (FD) transceiving, utilizing self-interference cancellation (SIC) to enhance PLS performance. Key operational parameters, including transmission probability (TxPr), friendly jammer density, and conditions for FD operation, are designed to maximize secrecy performance. The analytical and numerical results demonstrate significant improvements in PLS performance, with SIC playing a critical role, particularly in scenarios with dense legitimate nodes, and with TxPr adjusted to balance HD receiving and FD transceiving based on SIC imperfections. The proposed design principles provide a comprehensive framework for enhancing the security of WMB networks, addressing the complex interplay of interference and SIC in various network configurations.

## 1. Introduction

With the anticipated arrival of sixth generation (6G) technology, a highly intelligent society and lifestyle are on the horizon, driven by extensive wireless information exchanges between massive devices including sensors, Internet of Things (IoT) devices, mobile phones, and vehicles [1]. This proliferation of devices is expected to accelerate exponentially [2]. In particular, data sharing between legitimate yet unspecified neighboring nodes not only facilitates the detection of nearby devices [3,4,5] but also serves as a foundation for advanced proximity-based services. These services encompass a wide range of emerging applications, including geographic content sharing between nearby users or IoT devices [6], user- or device-aware services using Bluetooth low energy (BLE) beacons [7], social community detection in mobile social networks [8], and dissemination of driving safety information in vehicle-to-vehicle (V2V) networks [9,10]. This process, termed “wireless mutual broadcast (WMB)” [11] in this study, typically begins by broadcasting information to unspecified devices and gathering data from those in close proximity. However, this WMB is inherently vulnerable to eavesdropping because a transmitter ingenuously disseminates its broadcast message (BM) to unknown nodes. On the other hand, physical layer security (PLS) ensures nonzero secrecy capacity by maintaining the link quality of eavesdroppers below that of legitimate nodes [12], gaining significant attention as an effective alternative or complement to traditional cryptographic methods [13,14]. This approach is particularly useful in large-scale distributed networks where managing and sharing security keys may be challenging [14,15]. Recently, its applications have been being extended to various networks, including satellite–terrestrial integrated networks [16], simultaneous wireless information and power transfer networks [17], and covert mmWave communication networks [18]. From this perspective, this paper conducts an analytical exploration of the spatial PLS performances of the WMB.

In PLS, for a nonzero secrecy capacity [12], the connection quality for unauthorized eavesdroppers (Eves) needs to be kept below the performance of legitimate receivers, and this strategy may require knowledge about nearby legitimate nodes [13,14]. However, a WMB operation often commences without such prior information, and this poses significant challenges in integrating WMB with PLS strategies [11]. The work of [11] investigated the PLS performances of a random-access-based WMB (RA-WMB) network with half-duplex (HD) nodes, where nodes probabilistically alternate between transmitting and receiving broadcast messages (BMs). Although the work of [11] attempted to quantify the contribution of PLS on RA-WMB, it is still questionable whether or not the PLS performance of WMB can be further improved, and if so, how it can be achieved. The works in [19,20,21,22,23] demonstrated that full-duplex (FD) jamming can contribute to enhancing network-wide PLS performance depending on the self-interference cancellation (SIC) capability. This benefit motivates us to investigate the potential of SIC and to examine mediating HD and FD, in terms of enhancing the PLS performance of WMB.

### 1.1. Related Works

The data broadcast between neighbor nodes, i.e., WMB, supports a wide range of applications, including ambient data sharing [1,6], mobile social networking [8], intelligent transportation [9,10,24], and emergency communications [25,26]. Like any wireless network with performance limited by mutual interference [27], interference in WMB networks similarly impairs system performance. Stochastic geometry facilitates the mathematical tractability of wireless network performances through stochastically modeling the spatial radio interaction among devices [27], and several literatures have quantified and improved network-wide performances of WMB [24,28,29,30,31,32,33,34,35,36], under stochastic geometry frameworks. The works in [28,29] mathematically quantified the average number of successfully discovered nodes, and they proposed the design principles for improving performance. The peer discovery is a representative example of WMB when a BM is used for proximity awareness. The work in [31] studied the resource allocation method to mitigate interference through geo-location-based access in V2V WMB networks, and it demonstrated the performance gain through modeling vehicular networks based on stochastic geometry. The study on WMB has been further extended in conjunction with various issues including low power design [32], full duplex [33,34], and energy harvesting [35], in terms of network-wide performances. The works in [33,34] demonstrated that SIC significantly affects RA-WMB performance depending on the level of residual self-interference (RSI). In particular, the work of [34] showed that adjusting the proportions of simultaneous transceiving nodes and receiving-only nodes may improve RA-WMB performance compared to a scenario where all nodes are simultaneously transceiving with SIC, especially at high RSI levels. Furthermore, the work of [36] analytically examined the use of device-to-device (D2D) multicast for emergency information dissemination of a flying central station. Furthermore, the work of [24] analytically designed the broadcast period of vehicles in cooperative vehicle systems where vehicles periodically broadcast such information as position and speed via random access, using stochastic geometry theory. However, all of those works did not deal with the PLS performances of WMB.

The PLS offers advantages in the context of wireless networks by leveraging the inherent characteristics of physical medium [13], thus providing security that is inherently robust against eavesdropping without the complexities and vulnerabilities associated with key management and algorithmic encryption in conventional cryptographic methods. This makes PLS particularly suitable for dynamic and resource-constrained environments, such as wireless sensor, IoT, V2V, and D2D networks [14,15]. In order to confound Eves more actively, a legitimate transmitter and friendly jammer send artificial noise (AN) for impairing Eves’ link [14,15,37]. However, AN may be rather harmful to other nearby legitimate nodes in wireless networks because jamming signals raise interference. In this regard, through applying stochastic geometry theory, network-wide secrecy performances have been analytically quantified in various scenarios, e.g., the millimeter wave ad hoc network [38], three dimensional IoT networks [39], covert communication in D2D networks [40], vehicular networks [41], and uncoordinated random jamming in wireless sensor networks [42]. Further, several studies investigated the effect of an FD jamming approach, where FD nodes receive informative data from their transmitter while simultaneously sending AN for confounding Eves [19,20], in terms of strengthening network-wide secrecy performances in wireless distributed networks [21,22], under stochastic geometry frameworks. In particular, the work of [22] demonstrated that FD jamming impacts PLS performance depending on RSI level and proposed a switchable FD/HD mode. Hence, they concentrated on enhancing security by impairing the connections to these Eves while preserving the quality of the link to an intended receiver. In contrast, the WMB studied in this paper experiences inevitable performance degradation due to jamming, because both receivers and eavesdroppers remain unknown. This lack of information significantly makes the PLS in WMB challenging. The work of [11] analytically investigated the spatial secrecy performance of RA-WMB networks and demonstrated that simply adjusting a fraction of transmitters, by controlling transmission probability (TxPr), improved PLS performance. However, it did not consider FD operations with SIC.

### 1.2. Contributions and Organization

This paper investigates the performance and design principles of the secure RA-WMB that uses SIC for enhancing PLS performance. In particular, this study analytically quantifies the impact of SIC, through adjusting the proportion of legitimate HD receiving (i.e., not using SIC) and FD transceiving (i.e., using SIC) nodes, and this mixture of HD receiving and FD transceiving is referred to as the *hybrid duplex* (*HBD*). Even though a more advanced access method, such as carrier sense multiple access (CSMA), can further improve the performance, for mathematical tractability of network-wide PLS performance, this paper focuses on exploring the PLS of the HBD RA-WMB, and more complex WMB issues will remain as future works. This secure RA-WMB can serve as a baseline for evaluating the performance of more advanced secure WMB systems. The key contributions of this paper are highlighted as follows:

1. *Comprehensive analysis of secrecy performance for HBD RA-WMB*: This study analytically expresses the PLS performance of the HBD RA-WMB and quantitatively elucidates its inherent properties regarding the spatial radio interaction among legitimate FD transceving and HD receiving nodes, Eves, and friendly jammers. In particular, the analysis and numerical results demonstrate that the significant differences in the secrecy performance between secure HBD RA-WMB and conventional approaches, such as nonsecure RA-WMB [28,29,34] and secure HD RA-WMB [11]. These conventional methods can be regarded as special cases within the scope of the comprehensive results investigated herein.
2. *Proposition of design principles for secure HBD RA-WMB*: This study proposes the design principles of the key operation parameters including TxPr and friendly jammer density, in terms of maximizing the secrecy performance. In particular, the TxPr determines the operation method of nodes, such as FD transceving and HD receiving, through comprehensively considering the imperfections of SIC and beneficial and harmful effects of interference, with the aim of enhancing the secrecy performance of WMB. The analytical expression of the TxPr in secure HBD operation design explicitly distinguishes itself from the conventional non-secure RA-WMB design. In contrast, it is demonstrated that friendly jammers are not quite beneficial.
3. *Quantification of required SIC capability*: This study endeavors to quantitatively examine the RSI condition in imperfect SIC that are required for FD transceiving to significantly enhance secrecy performance, through identifying the conditions for the FD optimality and superiority. *FD optimality* denotes that it is optimal for all nodes to work in FD, while *FD superiority* indicates that FD outperforms HD in terms of secrecy performance. The FD optimality and superiority are addressed in terms of Eves density as well as RSI amount and wireless channels. It is remarkably interesting that these conditions increasingly become loose as Eves becomes dense.

Based on the comprehensive findings presented in this paper, Table 1 provides a concise summary and comparison of the design principles of TxPr, which determines the operations of WMB nodes for various RSI levels ranging from 0 to *∞*, between secure and nonsecure RA-WMB approaches. Note that the HD performance in [11] is equivalent to the HBD performance with an infinite RSI level in the model analyzed in this paper. In this table, $\lambda_{B}$ and $\lambda_{E}$ denote the spatial densities of legitimate nodes and Eves, respectively, and $\nu_{B}^{*}$ and β denote the optimal proportion of legitimate nodes transceiving with SIC (i.e., TxPr) and the amount of RSI, respectively. (Hence, the proportion of legitimate nodes receiving without SIC becomes $1-\nu_{B}^{*}$. Furthermore, the properties of $\nu_{B}^{*}$ are inferred from its suboptimal values and numerical results.) Further, $\beta_{FO}$ and $\beta_{FS}$ denote the values of β required for the FD optimality and superiority, respectively. The notations are described in Table 2, which also lists the parameter values used for the numerical results in Section 6.

The remainder of this paper is organized as follows. Section 2 describes the system model for the HBD RA-WMB and defines the secrecy performance metric. Section 3 analytically quantifies the secrecy performance, which unveils the inherent properties of HBD RA-WMB and the impact of AN. Section 4 proposes the design method of the HBD RA-WMB in terms of improving the secrecy performance. Section 5 further elaborates on the condition for FD operation as a special yet typical duplex scenario. Then, Section 6 discusses the numerical results, and Section 7 concludes the paper.

*Notations*: E[X] denotes the expectation of random variable *X* and P[E] represents the probability of event *E*. I[Y=y] is an indicator function whose value is 1 if Y=y while becoming 0 otherwise.

## 2. Secrecy Performance Models for Hybrid Duplex RA-WMB Networks

This section describes the HBD RA-WMB models where each legitimate node independently chooses either HD receiving or FD transceiving with a designed probability and defines the network-wide secrecy performance.

### 2.1. System Model

This paper considers RA-WMB networks, where legitimate nodes broadcast and gather their BMs, while Eves attempt to overhear those legitimate BMs. This study assumes that Eves try to decode BMs without any interference cancellation and any collusion among Eves, and more advanced operations by Eves will remain as future works. In order to safeguard BMs against Eves, the RA-WMB network employs Wyner’s wiretap encoding [12]. This method sets the rates for a transmitted codeword and confidential BM at $R_{T}$ and $R_{S}$, respectively, with the difference $R_{E}≜R_{T}-R_{S}$ serving as the rate redundancy to ensure secrecy against Eves. Perfect secrecy is achieved if $R_{E}$ exceeds the capacity of the most capable Eve’s link. Given that BMs must be decodable by nearby unspecified legitimate nodes, the data rate for BMs is usually fixed, making it feasible to establish predetermined values for $R_{T}$ and $R_{S}$. As a result, a node can securely receive a BM provided that it avoids the following two types of outages [11,22]: (i) a *connection outage*, where a legitimate receiver’s capacity drops below $R_{T}$, and (ii) a *secrecy outage*, where the capacity of the most malicious Eve exceeds $R_{E}$. Further, the network may consider deploying jamming nodes that transmit artificial noise (AN) to degrade the link quality for Eves. However, the AN transmission of these friendly jammers affects all nodes because they lack information about legitimate receivers. As a result, the AN broadcast may have a negative effect on legitimate nodes as well as Eves. In order to construct an analytically tractable network model, it is assumed that legitimate nodes and Eves are spatially distributed according to homogeneous Poisson point processes (HPPPs) with density $\lambda_{B}$ and $\lambda_{E}$, respectively.

**Figure 1:**
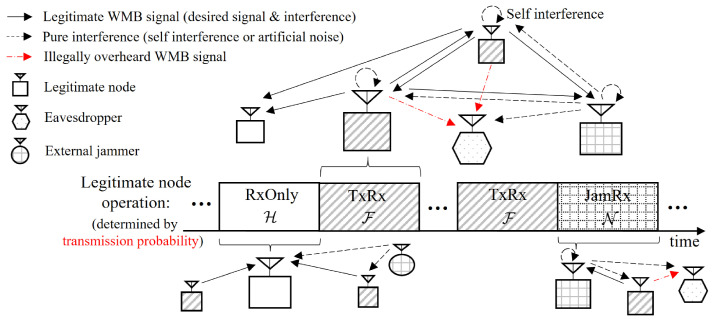
Operation models for secure HBD RA-WMB.

Figure 1 presents the operation model for the secure HBD RA-WMB. Each legitimate node either receives BMs from other nodes without SIC, or simultaneously transmits its own BM while receiving BMs from other nodes using SIC. The model assumes that all nodes are synchronized for this operation [28,29,33,35]. In this network, a legitimate node works in either of the following three modes:

(i) *RxOnly* (denoted by H): A node only gathers BMs broadcast by other legitimate nodes and it does not transmit any signal.
(ii) *TxRx* (denoted by F): A node transmits the BM that it generates as an information source, using transmit power *p*, and simultaneously attempts to decode BMs generated from other nodes through canceling the self-interference from its own transmission.
(iii) *JamRx* (denoted by N): A part of legitimate nodes transmit AN with transmit power $\delta_{N}p$ in order to confound unknown Eves, and they also attempt to receive BMs from other legitimate nodes employing SIC, similar to the TxRx mode.

Each node determines its operation mode in a probabilistic manner, that is, a legitimate node works in one mode of the RxOnly, TxRx, and JamRx with probability of $1-\nu_{B}-\nu_{N}$, $\nu_{B}$, and $\nu_{N}$. In particular, $\nu_{B}$ denotes the TxPr, which is a primary design parameter for the RA-WMB [11,28,29,34]. Note that $\nu_{B}$ and $\nu_{N}$ are constrained by $0<\nu_{B}\leq 1$, $0<\nu_{N}<1$, and $\nu_{B}+\nu_{N}\leq 1$. The TxRx and JamRx employ SIC, and FD operation can be readily modeled by setting $\nu_{B}$ to $1-\nu_{N}$. In particular, this paper also refers to the TxRx mode as *FD transceiving*. Further, the RxOnly mode, also known as *HD receiving*, does not utilize SIC. Original HD operation includes BM transmission without SIC in addition to RxOnly operation. Although these three operation modes for HBD do not exactly represent these HD operations due to the absence of a transmission-only mode, HBD can simulate HD performance when RSI is sufficiently large to fail to decode any BM in the TxRx and JamRx modes; thus, the HD results in [11] can be regarded as a specific instance of this study characterized by an excessively large RSI. As a result, the HBD with these three modes provides a comprehensive model of the duplexing methods, spanning from HD and FD to a pure HBD configuration where HD receiving and FD transceiving nodes coexist.

In the HBD RA-WMB networks, legitimate nodes in the JamRx mode are selected from all legitimate nodes and referred to as *internal jammers*. The network regulates the proportion of these internal jammers through adjusting $\nu_{N}$. Additionally, the system may employ *external jammers*, which do not possess any BM and solely transmit AN. External jammers are expected to be beneficial when interference only from legitimate nodes is insufficient to prevent Eves from overhearing BMs. It is assumed that these external jammers are also distributed according to an HPPP with density $\lambda_{J}$ and continuously send AN with transmit power $\delta_{J}p$ all the time.

The model considers wireless channels where a transmitted signal undergoes standard power loss propagation with path loss exponent α and Rayleigh fading with unit mean. When the spatial distribution of nodes follows an HPPP, the effects of independent and identically distributed shadowing can be expressed as another equivalent HPPP [28,43]; thus, the results in this paper can be straightforwardly extended to incorporate the impact of shadowing, similar to those in [28,29,34], even though this paper does not explicitly consider shadowing. The spatial node distributions of legitimate nodes, external jammers, and Eves are denoted by $\Phi_{B}$, $\Phi_{J}$, and $\Phi_{E}$, respectively, where $\Phi_{s}$ denotes an HPPP with density $\lambda_{s}$, where s∈{B,J,E}. Furthermore, β denotes the RSI normalized by *p*.

In this paper, $X_{l}$ denotes the location of node *l* that belongs to the set of legitimate nodes or external jammers, i.e., $X_{l}\in \Phi_{B}$ or $X_{l}\in \Phi_{J}$. In addition, $h_{li}$, *K*, and $\sigma^{2}$ denote the Rayleigh fading gain from nodes *i* to node *l*, the path loss gain at a unit distance, and $\frac{{\tilde{\sigma }}^{2}}{Kp}$ where ${\tilde{\sigma }}^{2}$ is noise power. When the typical node located at the origin works in mode *d* where d∈D≜{H,F,N}, the signal to interference plus noise ratio (SINR) of the link from a tagged node located at $X_{i}$ to the typical node is given by

(1) $$\begin{array}{c} \begin{array}{c} \Xi_{B}^{d}\left(X_{i}\right)≜\frac{h_{oi}{\left|X_{i}\right|}^{-\alpha }}{I_{B}+I_{J}+\beta_{d}/K+\sigma^{2}}, \end{array} \end{array}$$

where $I_{B}≜\sum_{X_{b}\in \Phi_{B}\setminus \left\{X_{i}\right\}}h_{ob}\left(I\left[d_{b}=F\right]+I\left[d_{b}=N\right]\delta_{N}\right){\left|X_{b}\right|}^{-\alpha }$, $I_{J}≜\sum_{X_{j}\in \Phi_{J}}h_{oj}\delta_{J}{\left|X_{j}\right|}^{-\alpha }$, and $d_{b}\in D$ means the operation mode of node *b*. It is noteworthy that $P\left[I\left[d_{b}=F\right]=1\right]\;=\;\nu_{B}$ and $P\left[I\left[d_{b}=N\right]=1\right]\;=\;\nu_{N}$. Further, $\beta_{d}≜\left(I\left[d=F\right]+I\left[d=N\right]\delta_{N}\right)\beta$, which models the effect of imperfect SIC in operation mode *d*.

On the other hand, an Eve located at $Y_{e}$ experiences the following SINR for node *i*:

(2) $$\begin{array}{c} \begin{array}{c} \Xi_{E}\left(X_{i},Y_{e}\right)≜\frac{h_{ei}{\left|X_{i}-Y_{e}\right|}^{-\alpha }}{I_{B,E}+I_{J,E}+\sigma^{2}}. \end{array} \end{array}$$

where $I_{B,E}≜\sum_{X_{b}\in \Phi_{B}\setminus \left\{X_{i}\right\}}h_{eb}\left(I\left[d_{b}=F\right]+I\left[d_{b}=N\right]\delta_{N}\right){\left|X_{b}-Y_{e}\right|}^{-\alpha }$ and $I_{J,E}≜\sum_{X_{j}\in \Phi_{J}}h_{ej}\delta_{J}{\left|X_{j}-Y_{e}\right|}^{-\alpha }$.

### 2.2. Network-Wide Secrecy Performance

In RA-WMB networks, because all legitimate nodes become an information source, performance can be measured by counting the number of successfully decoded BMs from arbitrary legitimate nodes [28,34], which is denoted by B. Unlike this B, secure RA-WMB networks have to count the number of BMs securely received at a legitimate node [11], which represents the BM that suffers from neither connection nor secrecy outage. This means the average number of the BMs that a legitimate node successfully decodes but all Eves fail to decode, which is denoted by $S_{o}$. A legitimate node receives BMs in either of H, F, or N, as described in Section 2.1, and as a result,

(3) $$\begin{array}{c} \begin{array}{c} S_{o}=\sum_{d\in D}\theta_{d}E\left[\sum_{X_{b}\in \Phi_{B}}I\left[d_{b}=F\right]P\left[\Xi_{B}^{d}\left(X_{b}\right)>\xi_{B}\right]P\left[\max_{Y_{e}\in \Phi_{E}}\Xi_{E}\left(X_{b},Y_{e}\right)\leq \xi_{E}\right]\right], \end{array} \end{array}$$

where $\theta_{H}≜1-\nu_{B}-\nu_{N}$, $\theta_{F}≜\nu_{B}$, $\theta_{N}≜\nu_{N}$. In (3), $\xi_{B}$ and $\xi_{E}$ represent the minimum SINR thresholds for a legitimate node’s and an Eve’s successful receiving, respectively. Note that nonsecure performance B is $S_{o}$ for $\lambda_{E}=0$ [28,34], and the secure performance only for HD (denoted by $S_{Ho}$) is equal to $S_{o}$ for β→∞ [11]. Further, when all legitimate nodes operate only in FD, this performance (denoted by $S_{Fo}$) becomes $S_{o}$ for $\nu_{B}=1-\nu_{N}$. Because legitimate receiving (Rx) nodes and Eves receive signals from the same transmitters, events $\left\{\Xi_{B}^{d}\left(X_{b}\right)>\xi_{B}\right\}$ and $\left\{\max_{Y_{e}\in \Phi_{E}}\Xi_{E}\left(X_{b},Y_{e}\right)\leq \xi_{E}\right\}$ are inherently correlated. This correlation further complicates the analysis of $S_{o}$. For ease of analysis, similar to the approach only for HD studied in [11], this paper approximates $S_{o}$ by disregarding the interference correlation, as follows:

(4) $$\begin{array}{c} \begin{array}{c} S_{o}\approx S≜\sum_{d\in D}\theta_{d}E\left[\sum_{X_{b}\in \Phi_{B}}I\left[d_{b}=F\right]P\left[\Xi_{B}^{d}\left(X_{i}\right)>\xi_{B}\right]\right]E\left[\prod_{Y_{e}\in \Phi_{E}}P\left[\Xi_{E}\left(X_{b},Y_{e}\right)\leq \xi_{E}\right]\right]. \end{array} \end{array}$$

Then, S in (6) also embraces the performances of nonsecure, only HD, and only FD operations as special cases, as follows:

(5) $$\begin{array}{c} \begin{array}{cc} \; & B\left(\nu_{B}\right)=S\left(\nu_{B},0,0\right){|}_{\lambda_{E}=0},\\ \; & S_{Ho}\approx S_{H}\left(\nu_{B},\nu_{N},\lambda_{J}\right)≜\lim_{\beta \to \infty }S\left(\nu_{B},\nu_{N},\lambda_{J}\right),\\ \; & S_{Fo}\approx S_{F}\left(\nu_{N},\lambda_{J}\right)≜S\left(1-\nu_{N},\nu_{N},\lambda_{J}\right). \end{array} \end{array}$$

The numerical and simulation results in Section 6 will demonstrate that $\left(S,S_{H},S_{F}\right)$ provide quite close approximations of $\left(S_{o},S_{Ho},S_{Fo}\right)$, even though these approximations exhibit small deviations from the original secrecy performances.

## 3. Spatial Secrecy Performance Analysis of Hybrid Duplex RA-WMB

This section investigates the secrecy performance and its inherent properties for secure HBD RA-WMB networks. The following result analytically expresses S in (4).

**Lemma** **1.** *When $\bar{\lambda }≜\lambda_{B}\nu_{B}+\lambda_{B}\nu_{N}\delta_{N}^{\frac{2}{\alpha }}+\lambda_{J}\delta_{J}^{\frac{2}{\alpha }}$,*

(6) $$\begin{array}{c} \begin{array}{c} S\left(\nu_{B},\nu_{N},\lambda_{J}\right)=\sum_{d\in D}\theta_{d}s_{d}\left(\nu_{B},\nu_{N},\lambda_{J}\right), \end{array} \end{array}$$

*where*

(7) $$\begin{array}{c} \begin{array}{c} s_{d}\left(\nu_{B},\nu_{N},\lambda_{J}\right)=\lambda_{B}\nu_{B}A_{d}\left(\bar{\lambda },\xi_{B}\right)\exp\left(-\lambda_{E}A_{H}\left(\bar{\lambda },\xi_{E}\right)\right), \end{array} \end{array}$$

(8) $$\begin{array}{c} \begin{array}{c} A_{d}\left(\lambda ,\xi \right)≜\int_{R^{2}}P\left[\Xi_{B}^{d}\left(x\right)>\xi_{B}\right]dx=\pi \int_{0}^{\infty }\exp\left(-\pi \lambda \xi^{\frac{2}{\alpha }}\Delta \left(\alpha \right)x-\xi \left(\beta_{d}/K+\sigma^{2}\right)x^{\frac{\alpha }{2}}\right)dx, \end{array} \end{array}$$

*$\Delta \left(\alpha \right)=\frac{2\pi /\alpha }{\sin(2\pi /\alpha )}$, and d∈D={H,F,N}.*

**Proof.**  See Appendix A. □

In Lemma 1, $A_{d}\left(\lambda ,\xi \right)$ can be interpreted as the area where a receiver can successfully decode BMs if transmitters are located therein when the SINR threshold is ξ. Therefore, $\theta_{d}s_{d}$ means the average number of the legitimate transmitting (Tx) nodes that are located in the receiving area of the typical node but not in the overhearing area of any Eve, like Figure 1.

### 3.1. Properties of Spatial Secrecy Performance

This subsection explores the inherent properties of S in (6).

**Proposition** **1.** *S in (6) has the following properties:*

(i) *When $\overset{ˇ}{S}≜\sum_{d\in D}\theta_{d}{\overset{ˇ}{s}}_{d}\left(\nu_{B},\nu_{N},\lambda_{J}\right)$ and $\tilde{S}≜\sum_{d\in D}\theta_{d}{\tilde{s}}_{d}\left(\nu_{B},\nu_{N},\lambda_{J}\right)$, $S>\overset{ˇ}{S}>\tilde{S}$ where*
(9) $$\begin{array}{c} \begin{array}{cc} {\overset{ˇ}{s}}_{d}\left(\nu_{B},\lambda_{J},\nu_{J}\right) & ≜\lambda_{B}\nu_{B}A_{d}\left(\bar{\lambda },\xi_{B}\right)\exp\left(-\lambda_{E}{\hat{A}}_{H}\left(\bar{\lambda },\xi_{E}\right)\right), \end{array} \end{array}$$
(10) $$\begin{array}{c} \begin{array}{c} {\tilde{s}}_{d}\left(\nu_{B},\lambda_{J},\nu_{J}\right)≜\frac{\lambda_{B}\nu_{B}}{\bar{\lambda }\xi_{B}^{2/\alpha }\Delta \left(\alpha \right)}\exp\left(-{\left(\pi \bar{\lambda }\Delta \left(\alpha \right)\right)}^{-\frac{\alpha }{2}}\Gamma \left(1+\frac{\alpha }{2}\right)\left(\beta_{d}/K+\sigma^{2}\right)-\lambda_{E}{\hat{A}}_{H}\left(\bar{\lambda },\xi_{E}\right)\right), \end{array} \end{array}$$
  *and ${\hat{A}}_{H}\left(\bar{\lambda },\xi \right)≜\frac{1}{\bar{\lambda }\xi^{\frac{2}{\alpha }}\Delta \left(\alpha \right)}$.*
(ii) *When β=0, in an interference-limited scenario,*
(11) $$\begin{array}{c} \begin{array}{cc} \lim_{\sigma^{2}\to 0}s_{d} & =\lim_{\sigma^{2}\to 0}{\overset{ˇ}{s}}_{d}=\lim_{\sigma^{2}\to 0}{\tilde{s}}_{d}=\frac{\lambda_{B}\nu_{B}}{\bar{\lambda }\xi_{B}^{2/\alpha }\Delta \left(\alpha \right)}\exp\left(-\lambda_{E}{\hat{A}}_{H}\left(\bar{\lambda },\xi_{E}\right)\right). \end{array} \end{array}$$
  *As a result,*
(12) $$\begin{array}{c} \begin{array}{cc} \lim_{\sigma^{2}\to 0}S & =\lim_{\sigma^{2}\to 0}\overset{ˇ}{S}=\lim_{\sigma^{2}\to 0}\tilde{S}=\frac{\lambda_{B}\nu_{B}}{\bar{\lambda }\xi_{B}^{2/\alpha }\Delta \left(\alpha \right)}\exp\left(-\lambda_{E}{\hat{A}}_{H}\left(\bar{\lambda },\xi_{E}\right)\right). \end{array} \end{array}$$
  *In particular, when $\nu_{N}$ and $\lambda_{J}$ are given, $\lim_{\sigma^{2}\to 0}S$ for β=0 is maximized at $\nu_{B}=1-\nu_{N}$.*
(iii) *Assuming that Eve’s receiver capability is not less than that of a legitimate node, i.e., $\xi_{E}\leq \xi_{B}$,*
(13) $$\begin{array}{c} \begin{array}{cc} \;S & \leq \lambda_{B}\nu_{B}A_{H}\left(\bar{\lambda },\xi_{B}\right)\exp\left(-\lambda_{E}A_{H}\left(\bar{\lambda },\xi_{E}\right)\right)\\ \; & \leq \lambda_{B}\nu_{B}A_{H}\left(\bar{\lambda },\xi_{B}\right)\exp\left(-\lambda_{E}A_{H}\left(\bar{\lambda },\xi_{B}\right)\right)\leq \hat{S}, \end{array} \end{array}$$
  *where $\hat{S}≜\frac{\lambda_{B}}{\lambda_{E}\exp\left(1\right)}$.*
(iv) *In a coverage-limited scenario without any interference,*
(14) $$\begin{array}{c} \begin{array}{c} S=\sum_{d\in D}\theta_{d}s_{d}\left(\nu_{B},\nu_{N},\lambda_{J}\right)=\sum_{d\in D}\theta_{d}\lambda_{B}\nu_{B}A_{d}\left(0,\xi_{B}\right)\exp\left(-\lambda_{E}A_{H}\left(0,\xi_{E}\right)\right), \end{array} \end{array}$$
  *where*
(15) $$\begin{array}{c} \begin{array}{c} A_{d}\left(0,\xi \right)=\pi \xi^{-\frac{2}{\alpha }}{\left(\frac{\beta_{d}}{K}+\sigma^{2}\right)}^{-\frac{2}{\alpha }}\Gamma \left(1+\frac{2}{\alpha }\right). \end{array} \end{array}$$
  *In particular, when $\nu_{N}=0$ and $\lambda_{J}=0$, $S(\nu_{B},0,0)$ is maximized at*
(16) $$\begin{array}{c} \begin{array}{c} \nu_{B}=\min\left\{1,\;\frac{A_{H}\left(0,\xi_{B}\right)}{2\left(A_{H}\left(0,\xi_{B}\right)-A_{F}\left(0,\xi_{B}\right)\right)}\right\}. \end{array} \end{array}$$
(v) *When $\nu_{N}=0$ and $\lambda_{J}=0$, there exists such $\beta_{A}>0$ that $S\left(1,0,0\right)\geq \max_{\nu_{B}}S_{H}\left(\nu_{B},0,0\right)$ as long as $\beta \leq \beta_{A}$.*

**Proof.**  See Appendix B. □

It is not straightforward to deal with S in (6), which includes the sum of numerical integrals. In this regard, Proposition 1-(i) and (iii) provide the lower and upper bounds of S that have further simplified forms. From Proposition 1-(ii), it is expected that the lower bound in Proposition 1-(i) becomes tighter as the interference becomes increasingly dominant. In particular, for an ideal SIC (i.e., β=0), these lower bounds become exactly the same as S. From these properties, for a small value of β and $\sigma^{2}$, it is expected that $\tilde{S}$ provides a tight lower bound of S. It is worth noting that the bounds in Proposition 1-(i) and (iii) serve as crucial tools for designing $\nu_{B}$ (to be discussed in Section 4.1) and for analyzing the influence of external jammers (to be addressed in Section 3.2).

In a coverage-limited scenario where the impact of interference is ignored, e.g., a sparse node distribution scenario, S can be expressed in a quite simple form like (14) and (15). Interestingly, Proposition 1-(iv) also gives the optimal $\nu_{B}$ for $\nu_{N}=0$ and $\lambda_{J}=0$, which does not depend on $\lambda_{E}$. This signifies that there is no difference between secure and nonsecure designs when legitimate nodes are sparse. Section 6 will demonstrate that secure design plays a more crucial role in improving secrecy performance as interference increases. However, designing $\nu_{B}$ in nonzero interference scenarios is not straightforward, which will be thoroughly addressed in Section 4.1. The optimal $\nu_{B}$ in (16) increases with $A_{F}\left(0,\xi_{B}\right)$, and this implies that the HBD RA-WMB moves toward the FD RA-WMB as RSI decreases. On the other hand, as β→∞, $A_{F}\left(0,\xi_{B}\right)$ goes to zero, and this leads that the optimal $\nu_{B}$ for HD RA-WMB becomes $\frac{1}{2}$. This result is consistent with the design in [29] that maximizes the nonsecure performance of HD RA-WMB.

Propositions 1-(v) indicate the *FD superiority*, which denotes that FD outperforms HD in terms of secrecy performance. This FD superiority significantly depends on RSI, and this issue will be addressed in more detail in Section 5.

### 3.2. Impact of Internal and External Jammers

To begin with, the following result demonstrates the *ineffectiveness of internal jammers*.

**Corollary** **1.** *An internal jamming signal does not contribute to improving S.*

**Proof.**  From (7), note that $s_{d}\leq {\overset{´}{s}}_{d}\left(\nu_{B},\nu_{N},\lambda_{J}\right)≜\lambda_{B}\left(\nu_{B}+\nu_{N}\right)A_{d}\left(\bar{\lambda },\xi_{B}\right)\exp\left(-\lambda_{E}A_{H}\left(\bar{\lambda },\xi_{E}\right)\right)$, where ${\overset{´}{s}}_{d}$ means that internal jammers do not send an AN signal but transmit their BM with transmit power $\delta_{N}p$. □

From Corollary 1, the maximization of S can be achieved through adjusting $\nu_{B}$ and $\lambda_{J}$ for $\nu_{N}=0$. This result is consistent with the result in Corollary 3.1 in [11] for HD. From now, if not stated otherwise, *this paper assumes that $\nu_{N}=0$ from Corollary 1*.

The following result addresses the contribution of the external jammers.

**Corollary** **2.** *An external jamming signal may contribute to increasing S. In particular, $\lambda_{J}>0$ is useful for a high Eve density. For example, in an interference-limited scenario with β=0 and $\xi_{B}=\xi_{E}$, when $\lambda_{E}>\lambda_{B}\xi^{\frac{2}{\alpha }}\Delta \left(\alpha \right)$, S is maximized at $\nu_{B}=1$ and $\lambda_{J}={\bar{\lambda }}_{J}$, where ${\bar{\lambda }}_{J}≜\left(\frac{\lambda_{E}}{\xi^{\frac{2}{\alpha }}\Delta \left(\alpha \right)}-\lambda_{B}\right)\delta_{J}^{-\frac{2}{\alpha }}$.*

**Proof.**  When β=0 and $\xi_{B}=\xi_{E}$, from (13), $S\leq \hat{S}$. Through setting $\left(\nu_{B},\lambda_{J}\right)$ to $\left(1,{\bar{\lambda }}_{J}\right)$, S achieves $\max_{0<\nu_{B}\leq 1}\hat{S}=\frac{\lambda_{B}}{\lambda_{E}\exp\left(1\right)}$. □

From the above result, this study continues to explore scenarios where $\lambda_{J}\geq 0$. Further, it is worth noting that Corollary 2 offers the results for HBD including FD, while Corollary 3.1 in [11] was only for HD.

## 4. Design Principles for Secrecy Performance Improvement in RA-WMB Networks

Although Section 3 highlights the intriguing properties of the secure HBD RA-WMB, the results do not clearly delineate the impact of key parameters such as TxPr and RSI on the secrecy performance, because S in (6) remains complex. Therefore, this section further explicitly quantifies the properties of S through approximating the secrecy performance and proposes the design methods of the key parameters, including $\nu_{B}$, β, and $\lambda_{J}$.

### 4.1. Tx Probability Design for Secure RA-WMB

As clarified in Section 3.2, internal jamming does not contribute to increasing S while external jamming may be helpful for high $\lambda_{E}$. In other words, the design can be simplified through setting $\nu_{N}$ to 0, and it is also expected that $\lambda_{J}=0$ is sensible if the Eve density is not quite large. Hence, this subsection focuses on the design of $\nu_{B}$ for increasing S when $\nu_{N}=0$ and $\lambda_{J}=0$. When defining the optimal $\nu_{B}$ for a given $\lambda_{J}$ as

(17) $$\begin{array}{c} \begin{array}{c} \nu_{B}^{*}\left(\lambda_{J}\right)≜\arg\max_{0<\nu_{B}\leq 1}S, \end{array} \end{array}$$

the design objective in this subsection is to approximate $\nu_{B}^{*}\left(0\right)$. The following results provide the fundamentals on the design of $\nu_{B}$ through examining $\tilde{S}$ in Proposition 1-(i).

**Proposition** **2.** *Assume that $\nu_{N}=0$ and $\lambda_{J}=0$. Furthermore, let ${\tilde{\nu }}_{B}≜\arg\max_{0<\nu_{B}\leq 1}\tilde{S}\left(\nu_{B},0,0\right)$, $\Theta ≜\frac{1}{\xi_{B}^{2/\alpha }\Delta \left(\alpha \right)}$, $\Omega ≜\frac{\alpha }{2}{\left(\pi \lambda_{B}\Delta \left(\alpha \right)\right)}^{-\alpha /2}\Gamma \left(1+\frac{\alpha }{2}\right)$, and $\Lambda_{E}≜\frac{\lambda_{E}}{\lambda_{B}\xi_{E}^{2/\alpha }\Delta \left(\alpha \right)}$. Then, $\tilde{S}\left(\nu_{B},0,0\right)$ have the following properties:*

(i) *There exists a $\beta_{c}>0$ such that $\tilde{S}\left(\nu_{B},0,0\right)$ monotonically increases with $\nu_{B}$ if $\beta <\beta_{c}$. That is, if $\beta <\beta_{c}$, ${\tilde{\nu }}_{B}=1$.*
(ii) *If β→∞, $\tilde{S}\left(\nu_{B},0,0\right)$ is log-concave for $0<\nu_{B}\leq 1$, and $\lim_{\beta \to \infty }{\tilde{\nu }}_{B}$ becomes ${\bar{\nu }}_{\infty }$ or 1, where ${\bar{\nu }}_{\infty }$ is the unique solution of $f_{{\bar{\nu }}_{\infty }}\left(x\right)=0$ if there exists a solution of $f_{\bar{\nu },\infty }\left(x\right)=0$ for 0<x<1, and*
(18) $$\begin{array}{c} \begin{array}{cc} f_{\bar{\nu },\infty }\left(x\right) & ≜x^{\frac{\alpha }{2}+1}+\Lambda_{E}x^{\frac{\alpha }{2}}-\Lambda_{E}x^{\frac{\alpha }{2}-1}+\Omega \sigma^{2}x-\Omega \sigma^{2}. \end{array} \end{array}$$
(iii) *For a finite β>0, ${\tilde{\nu }}_{B}\in {\tilde{N}}_{\nu_{B}}$ or 1, where ${\tilde{N}}_{\nu_{B}}≜\left\{x\;|\;f_{{\tilde{\nu }}_{B}}\left(x\right)=0\;for\;0<x\leq 1\right\}$, and $f_{{\tilde{\nu }}_{B}}\left(x\right)$ is defined in *(A10).

**Proof.**  See Appendix C. □

Proposition 2-(i) means that FD operation with $\nu_{B}=1$ is recommended for RSI below a certain level. Furthermore, Proposition 2-(ii) derives $\nu_{B}$ to maximize $\tilde{S}$ for an infinite RSI, which serves as the lower bound of the secrecy performance of HD RA-WMB. This result aligns with the result in Proposition 3.1 of [11]. Proposition 2-(iii) provides a necessary condition for ${\tilde{\nu }}_{B}$, but it is not easy to derive ${\tilde{\nu }}_{B}$ for general β when considering that $f_{{\tilde{\nu }}_{B}}\left(x\right)$ in (A10) has a complex form.

Therefore, this paper devises the design method of $\nu_{B}$ through comprehensively considering the following important values of $\nu_{B}$ in terms of maximizing S:

- $\nu_{B}=1$: FD optimality from Propositions 1-(ii) and 2-(i) and the FD superiority from Proposition 1-(v).
- $\nu_{B}={\bar{\nu }}_{\infty }$: ease of deriving ${\tilde{\nu }}_{B}$ for HD RA-WMB from Proposition 2-(ii) [11]
- $\nu_{B}=\frac{1}{2}$: importance of attentive listening from Proposition 1-(iv) and [29].

As a result, when $\nu_{N}=0$ and $\lambda_{J}=0$, the design method for $\nu_{B}$ is proposed in terms of increasing S, as follows:

(19) $$\begin{array}{c} \begin{array}{c} {\overset{˘}{\nu }}_{B}≜\arg\max_{\nu_{B}\in {\overset{˘}{N}}_{{\tilde{\nu }}_{B}}}S\left(\nu_{B},0,0\right), \end{array} \end{array}$$

where ${\overset{˘}{N}}_{{\tilde{\nu }}_{B}}≜\left\{{\bar{\nu }}_{\infty },\frac{1}{2},1\right\}$, and $S({\bar{\nu }}_{\infty },0,0)=-\infty$ if ${\bar{\nu }}_{\infty }$ does not exist.

### 4.2. FD Optimality for Secure RA-WMB without Any Jamming Signal

This subsection focuses on the condition for $\nu_{B}^{*}\left(0\right)=1$. This FD optimality condition can be expressed in terms of RSI, but it is not straightforward to derive this condition directly from S in (6).

The following result gives the FD optimality condition in an extreme scenario where the effect of interference is ignored.

**Corollary** **3.** In a coverage-limited scenario without any interference, $\nu_{B}^{*}\left(0\right)$ becomes one if

(20) $$\begin{array}{c} \begin{array}{c} A_{F}\left(0,\xi_{B}\right)\geq \frac{1}{2}A_{H}\left(0,\xi_{B}\right), \end{array} \end{array}$$

*which is equivalent to*

(21) $$\begin{array}{c} \begin{array}{c} \beta \leq \beta_{FO,0}≜K\sigma^{2}\left(2^{\frac{\alpha }{2}}-1\right). \end{array} \end{array}$$

**Proof.**  From (16), note that if $\frac{A_{H}\left(0,\xi_{B}\right)}{2\left(A_{H}\left(0,\xi_{B}\right)-A_{F}\left(0,\xi_{B}\right)\right)}\geq 1$, $\nu^{*}$ becomes one. From this FD optimality condition and (15), (20) and (21) are derived. □

It is worth noting that the performance becomes less sensitive to β as interference increases from (1); hence, it is sensible to consider $\beta_{FO,0}$ in (21) as the lowest RSI threshold for the FD optimality. In other words, for the FD optimality, RSI does not need to be below $\beta_{FO,0}$.

For a general scenario with nonzero interference, it is not easy to express the FD optimality condition analytically. It is highly likely that ${\overset{˘}{\nu }}_{B}$ in (19) is a nonincreasing function of β because simultaneous transceiving using SIC is increasingly preferred as β decreases, which can be demonstrated through the numerical results. As a result, the FD optimality condition is approximated as

(22) $$\begin{array}{c} \begin{array}{c} \beta \leq \beta_{FO}≜\max_{\beta \in \{\beta \;|\;{\overset{˘}{\nu }}_{B}=1\}}\beta , \end{array} \end{array}$$

where $\beta_{FO}$ can be found between $\beta_{FO,0}$ and a certain upper limit (e.g., −100dB) for a given HBD RA-WMB environment, via the bisection method, from Proposition 2-(i) and the nonincreasing property of ${\overset{˘}{\nu }}_{B}$ with respect to β.

### 4.3. Deployment of External Jamming Nodes for Secure RA-WMB

Corollary 2 demonstrated that $\lambda_{J}>0$ may contribute to increasing S for a high $\lambda_{E}$. In other words, when the amount of interference from legitimate nodes is not large enough to prevent Eves from overhearing BMs, the external jamming further raises interference for disturbing Eves’ reception. In contrast, $\lambda_{J}>0$ also degrades the legitimate link quality because of the unspecified radiation of jamming signals. As a result, it seems to be sensible that the increase in $\nu_{B}$ is first preferred to the increase in $\lambda_{J}$. Therefore, this paper designs $\lambda_{J}>0$ for increasing S, through fixing $\nu_{B}$ to one. The following result suggests the value of $\lambda_{J}$ for $\nu_{B}=1$ through approximating S as $\tilde{S}$.

**Proposition** **3.** *Assume that $\nu_{B}=1$ and $\nu_{N}=0$. Then, $\tilde{S}\left(1,0,\lambda_{J}\right)$ can be maximized at*

(23) $$\begin{array}{c} \begin{array}{c} \lambda_{J}={\hat{\lambda }}_{J,F}≜\lambda_{B}\delta_{J}^{-\frac{2}{\alpha }}\left(\frac{1}{\hat{x}}-1\right),if\;\lambda_{E}\geq {\bar{\lambda }}_{E,F}≜\lambda_{B}\xi_{E}^{2/\alpha }\Delta \left(\alpha \right)\left(1-\Omega \left(\frac{\beta }{K}+\sigma^{2}\right)\right), \end{array} \end{array}$$

*where $\hat{x}$ is the unique solution of $f_{J}\left(x\right)$ = 0,*

(24) $$\begin{array}{c} \begin{array}{cc} f_{J}\left(x\right) & ≜\Omega \left(\frac{\beta }{K}+\sigma^{2}\right)x^{\frac{\alpha }{2}}+\Lambda_{E}x-1, \end{array} \end{array}$$

*and $\Lambda_{E}$ and Ω are defined in Proposition 2.*

**Proof.**  See Appendix D. □

However, because $\tilde{S}$ deviates from S for a non-small β and $\sigma^{2}$, the design of $\lambda_{J}$ in Proposition 3 works well only for a small β. Then, one has a question about how to set $\lambda_{J}$ more accurately. The numerical results in Section 6 demonstrate that $\lambda_{J}$ primarily contributes to the increase in S only when Eves are dense, e.g., $\lambda_{E}>\lambda_{B}$. This excessive Eve density makes secure WMB operation difficult, and one should avoid this scenario. Further, the contribution of $\lambda_{J}$ is quite marginal, which will be presented via the numerical results in Section 6.

## 5. Design Principles of Secure RA-WMB for Full Duplex Case

The analytical exploration of S in Section 3 and Section 4 is for the HBD operations that are a mixture of HD and FD modes. Hence, this section discusses a specific but useful extreme case with $\nu_{B}=1$, where all legitimate nodes work in FD mode. For the other extreme scenario, where β→∞ and all legitimate nodes operates in HD mode, the relevant findings were detailed in [11], which can be regarded as a specific instance of the broader results discussed in this paper. From the assumption of $\nu_{N}=0$ as a result of Corollary 1, the secrecy performance of the FD RA-WMB in (5) can be recast into

(25) $$\begin{array}{c} \begin{array}{c} S_{F}\left(\lambda_{J}\right)≜S\left(1,0,\lambda_{J}\right)=s_{F}\left(1,0,\lambda_{J}\right). \end{array} \end{array}$$

The impact of external jamming signals has been already clarified in Corollary 2 because they include the case of $\nu_{B}=1$, that is, $\lambda_{J}>0$ marginally contributes to improving $S_{F}\left(\nu_{N},\lambda_{J}\right)$ for a large $\lambda_{E}$. Therefore, this subsection further focuses on the FD superiority stated in Section 3.1 through setting $\lambda_{J}$ to 0, which is given by

(26) $$\begin{array}{c} \begin{array}{c} S_{F}\left(0\right)>\max_{0<\nu_{B}<1}S_{H}\left(\nu_{B},0,0\right). \end{array} \end{array}$$

This FD superiority is a basic motivation of applying SIC in order to enhance the secrecy performance.

In a coverage-limited scenario, the FD superiority condition is expressed laconically as follows.

**Corollary** **4.** *In a coverage-limited scenario, the FD superiority condition is*

(27) $$\begin{array}{c} \begin{array}{c} A_{F}\left(0,\xi_{B}\right)>\frac{1}{4}A_{H}\left(0,\xi_{B}\right). \end{array} \end{array}$$

*This condition is equivalent to*

(28) $$\begin{array}{c} \begin{array}{c} \beta \leq \beta_{FS,0}≜K\sigma^{2}\left(2^{\alpha }-1\right). \end{array} \end{array}$$

**Proof.**  Because $\lim_{\beta \to \infty }A_{F}\left(0,\xi_{B}\right)=0$ from (16), $\max_{\nu_{B}}S_{H}\left(\nu_{B},0,0\right)=S_{H}\left(\frac{1}{2},0,0\right)$. Hence, from (26), $S_{F}\left(0\right)>S_{H}\left(\frac{1}{2},0,0\right)$. Through substituting (14) into this condition, (27) is obtained. Finally, from (15) and (27), (28) is derived. □

Note that $\beta_{FS,0}$ in (28) is larger than $\beta_{FO,0}$ in (21). Even though a coverage-limited scenario is an extreme scenario, this result explicitly reveals the difference between the FD optimality and superiority.

In a general scenario with nonzero interference, it is not easy to analytically express the condition of RSI for the FD superiority. Therefore, through approximating $\max_{0<\nu_{B}<1}S_{H}\left(\nu_{B},0,0\right)$ as $S_{H}\left({\overset{˘}{\nu }}_{\infty },0,0\right)$, (26) is now replaced with the following equation:

(29) $$\begin{array}{c} \begin{array}{c} S_{F}\left(0\right)>S_{H}\left({\bar{\nu }}_{\infty },0,0\right). \end{array} \end{array}$$

For FD superiority, when defining $\beta_{FS}$ as

(30) $$\begin{array}{c} \begin{array}{c} \beta_{FS}≜\beta \;\mathrm{such}\;\mathrm{that}\;S_{F}\left(0\right)=S_{H}\left({\bar{\nu }}_{\infty },0,0\right), \end{array} \end{array}$$

this $\beta_{FS}$ can be numerically found via the bisection method because $S_{F}\left(0\right)$ is a monotonic decreasing function of β. It is expected that $\beta_{FS}>\beta_{FO}$, where $\beta_{FO}$ was defined in (22), because the FD superiority is a more relaxed condition compared with the FD optimality. Further, when considering that the impact of β becomes less dominant as interference increases, it is expected that $\beta_{FO,0}<\beta_{FO}$ and $\beta_{FS,0}<\beta_{FS}$.

## 6. Numerical Results and Discussion

This section numerically evaluates and discusses the spatial secrecy performances of the HBD RA-WMB and its design methods that were investigated in the previous sections. The evaluation models comply with the system model described in Section 2, and if not stated otherwise, the system parameters are set to the values presented in Table 2.

**Figure 2:**
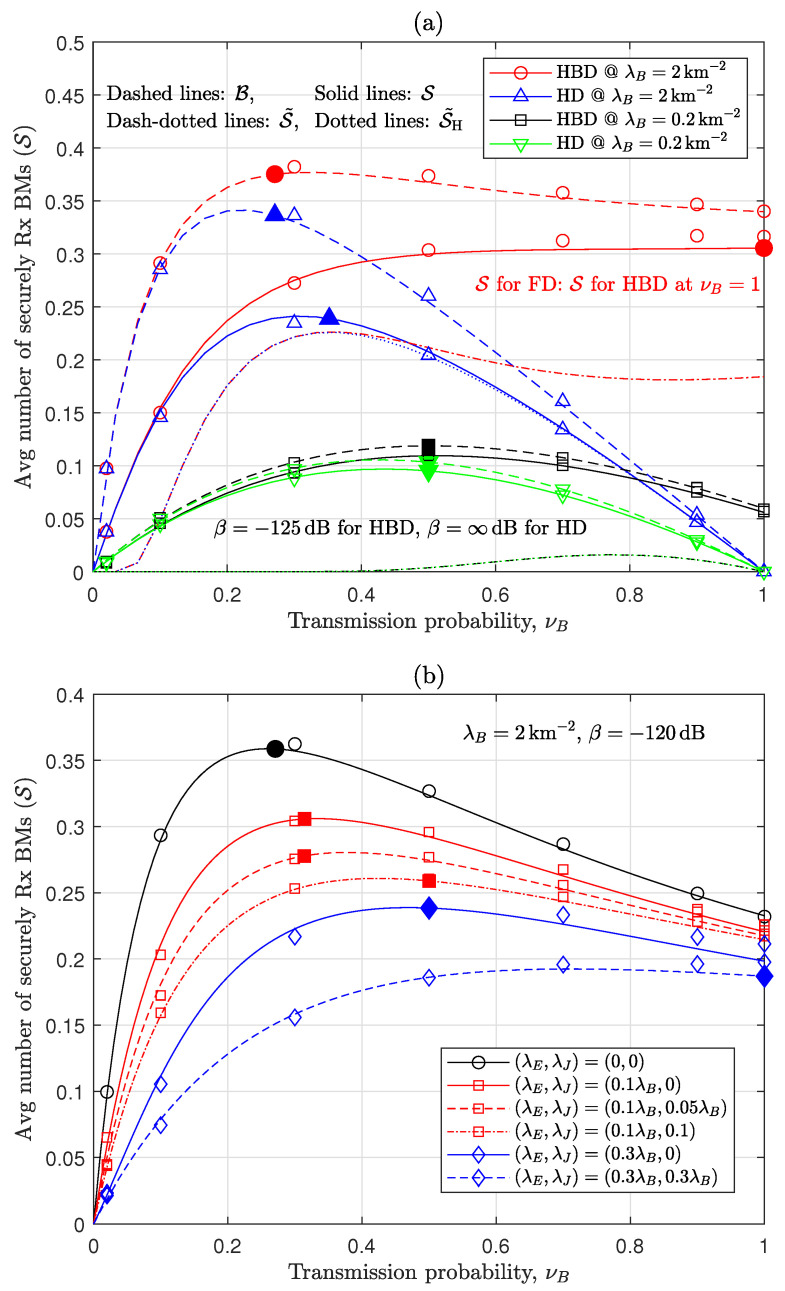
Properties of S [lines: analysis; open symbols: simulation ($S_{o}$); solid symbols: suboptimal $\nu_{B}$ given by ${\overset{˘}{\nu }}_{B}$ in (19)]: (**a**) S and its approximations ($\lambda_{E}=0.2\lambda_{B}$, $\lambda_{J}=0$). (**b**) Impact of Eves and external jammers.

Figure 2 exhibits the properties of S analyzed in Section 3.1. To begin with, the results demonstrate that S ignoring interference correlation agrees well with the simulation results that exactly reflect correlated interference. In Figure 2a, it is evident that Eves degrade S from S<B and HBD outperforms HD. When examining the performances of HBD at $\nu_{B}=1$, which denotes FD operation, FD is preferable at a higher $\lambda_{B}$, while a pure HBD strategy incorporating both HD receiving (i.e., in the RxOnly mode) and FD transceiving (i.e., in the TxRx mode) nodes is more advantageous at a lower $\lambda_{B}$. Further, ${\overset{˘}{\nu }}_{B}$ in (19) traces quite well the peak point of S in various interference scenarios (including both small and large impacts of $\sigma^{2}$), even though $\tilde{S}$ deviates from S. In particular, when $\lambda_{B}=2\;{\mathrm{km}}^{-2}$, Figure 2a presents that the optimal operation for B is a mixture of FD transceiving and HD receiving with ${\overset{˘}{\nu }}_{B}=0.27$, while the maximization of S for $\lambda_{E}=0.2\lambda_{B}$ is achieved at FD operation with $\nu_{B}=1$, and this implies that Eves significantly affect the optimal TxPr design. In contrast, as expected in Proposition 1-(iv), in scenarios with sparse legitimate nodes and Eves (e.g., $\lambda_{B}=0.2\;{\mathrm{km}}^{-2}$, $\lambda_{E}=0.04\;{\mathrm{km}}^{-2}$), the impact of Eves on the TxPr design becomes relatively smaller. In Figure 2a, because the effect of β=−125dB is small for $\lambda_{B}=2\;{\mathrm{km}}^{-2}$ while it is large for $\lambda_{B}=0.2\;{\mathrm{km}}^{-2}$; thus, the optimal TxPr for $\lambda_{B}=0.2\;{\mathrm{km}}^{-2}$ is around $\frac{1}{2}$. On the other hand, Figure 2b further explicitly reveals the significant impact of $\lambda_{E}$ on the optimal TxPr. It signifies that as Eves becomes denser, the legitimate nodes needs to generate more interference for confounding Eves. Further, Figure 2b presents that $\lambda_{J}$ affects the TxPr design, where ${\overset{˘}{\nu }}_{B}$ in (19) was obtained using ${\bar{\nu }}_{\infty }$ derived from $f_{\bar{\nu },\infty ,J}\left(\nu_{B}\right)=0$ in (A11) for $\lambda_{J}>0$. In this subfigure, $\lambda_{J}$ degrades S and the optimization and impact of this $\lambda_{J}$ will be presented and discussed in Figure 6 in more detail.

**Figure 3:**
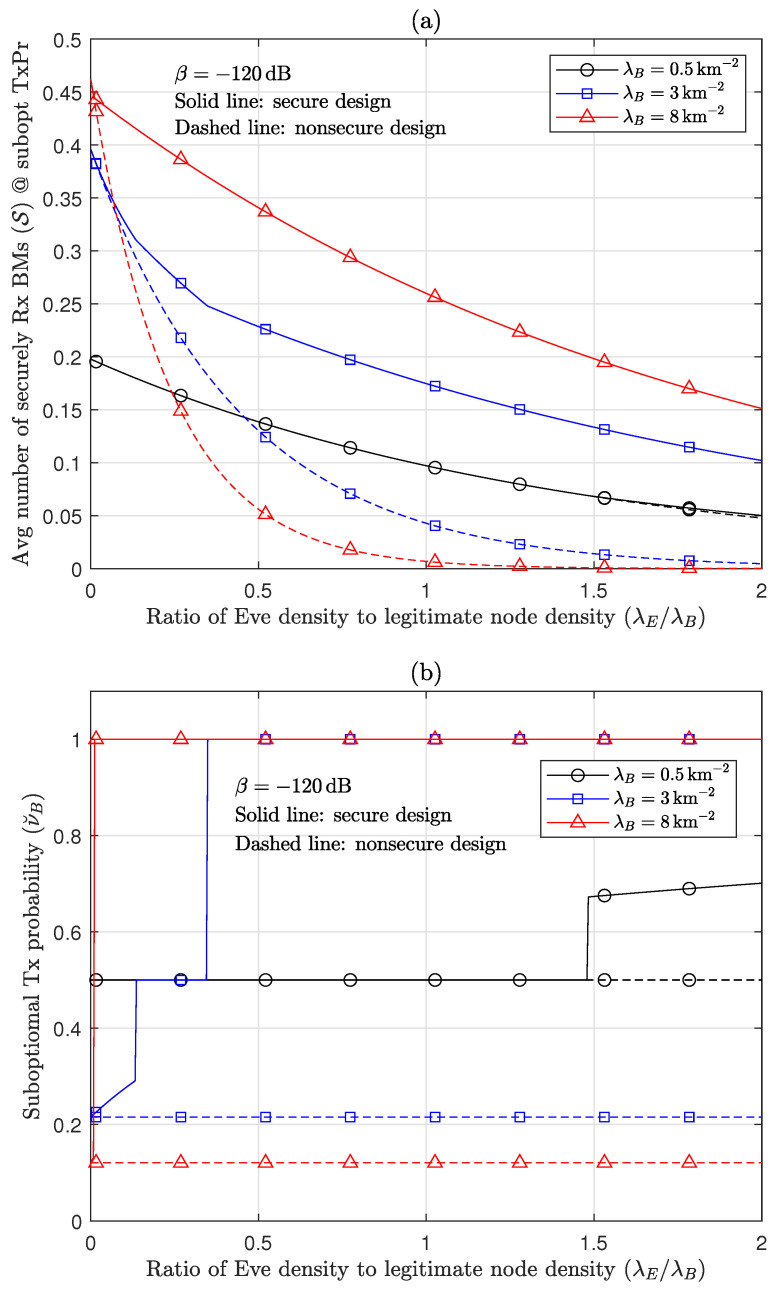
Comparison of secure and nonsecure designs for HBD RA-WMB: (**a**) Effect of Eve density on suboptimal S. (**b**) Effect of Eve density on suboptimal $\nu_{B}$.

Figure 3 demonstrates the effect and motivation of the secure design in the RA-WMB network. When TxPrs are securely and nonsecurely designed to increase S and B according to (19), respectively, Figure 3a compares the secrecy performances of the two designs. In the nonsecure design, recall that the TxPr for increasing B can be derived through setting $\lambda_{E}$ to zero in (19), and the value of S for this TxPr is measured. In the interference-dominant scenario with $\lambda_{B}=8\;{\mathrm{km}}^{-2}$, the secure design is outstandingly superior to the nonsecure one. It is interesting that the proposed secure design still significantly improves the secrecy performance even when legitimate nodes are not quite dense, e.g., when $\lambda_{B}=3\;{\mathrm{km}}^{-2}$. The nonsecure design increasingly degrades the secrecy performance as legitimate nodes become dense because the nonsecure design tends to decrease TxPr for mitigating interference [28,29,34] even if interference needs to be increased to confound Eves. Figure 3b clearly exhibits the difference between these TxPr designs. As $\lambda_{B}$ increases, the secure design more aggressively transmits BMs through increasing $\nu_{B}$, while the nonsecure design becomes more silent through decreasing $\nu_{B}$. Further, the nonsecure design does not respond to the increase in Eve density. In contrast, the secure design more rapidly reaches the FD operation as Eves become dense. This signifies that SIC can play a key role in increasing S as well as improving B [34].

**Figure 4:**
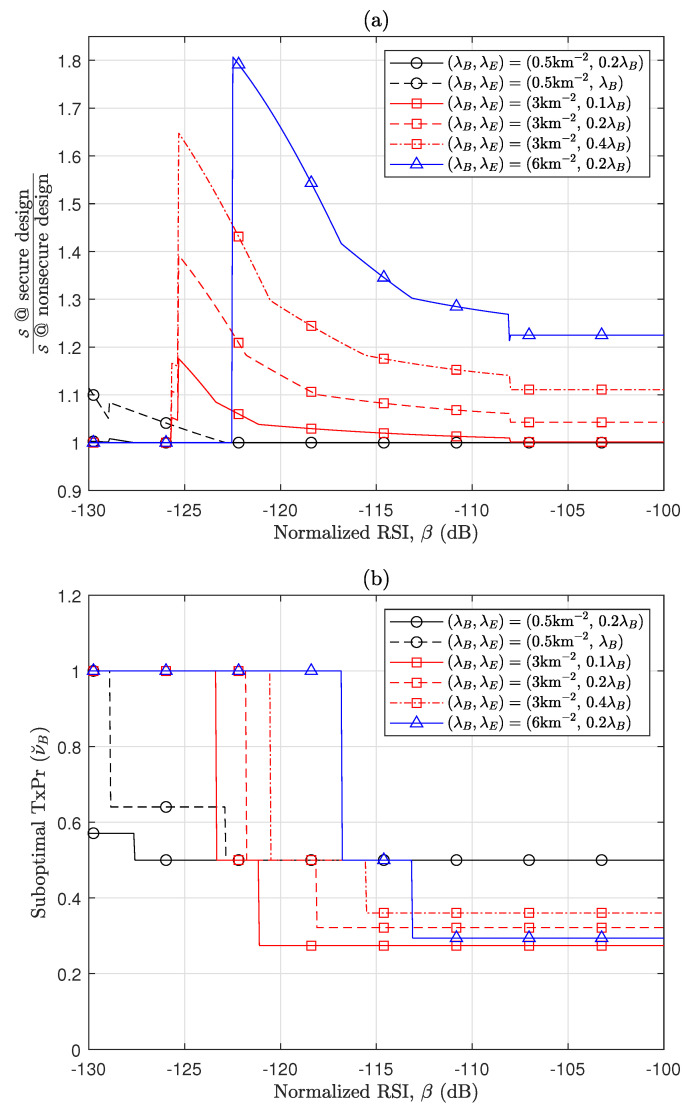
Contribution of SIC capability to secure RA-WMB (**a**) Effect of RSI on gain of secure RA-WMB design. (**b**) Effect of RSI on suboptimal $\nu_{B}$.

Figure 4 quantitatively presents the contribution of SIC to the improvement of S and the design of TxPr, through parameterizing the RSI level. In Figure 4a, the *y*-axis denotes the gain of the secure design with respect to the nonsecure design, i.e., $\frac{S({\overset{˘}{\nu }}_{B},0,0)}{S({\overset{˘}{\nu }}_{B}{|}_{\lambda_{E}=0},0,0)}$, when $\nu_{B}$’s are designed to increase S and B, respectively. In the scenarios with $\lambda_{B}=3\;{\mathrm{km}}^{-2}$, which represent scenarios where legitimate nodes are not quite dense, the secure design provides a significant gain even when Eves are not dense, e.g., about 3–18 % for β=−115dB. This gain substantially increases as RSI decreases, e.g., S increases by 17–64% when β=−125dB. As interference becomes more dominant, the gain becomes dramatically large. Further, it is interesting that even when legitimate nodes are sparse (e.g., when $\lambda_{B}=0.5\;{\mathrm{km}}^{-2}$), the secure design is beneficial if low RSI is implemented. On the other hand, Figure 4b exhibits the impact of RSI on the suboptimal TxPr for secure designs in the same scenarios, demonstrating that the coexistence of nodes in the RxOnly and TxRx modes becomes preferable over scenarios where all nodes operate exclusively in the TxRx mode, as RSI increases. This highlights that when the SIC capability is not excellent, balancing the beneficial and harmful effects of interference is more important than simply maximizing the simultaneous transceiving of individual nodes through SIC, due to non-negligible self-interference.

**Figure 5:**
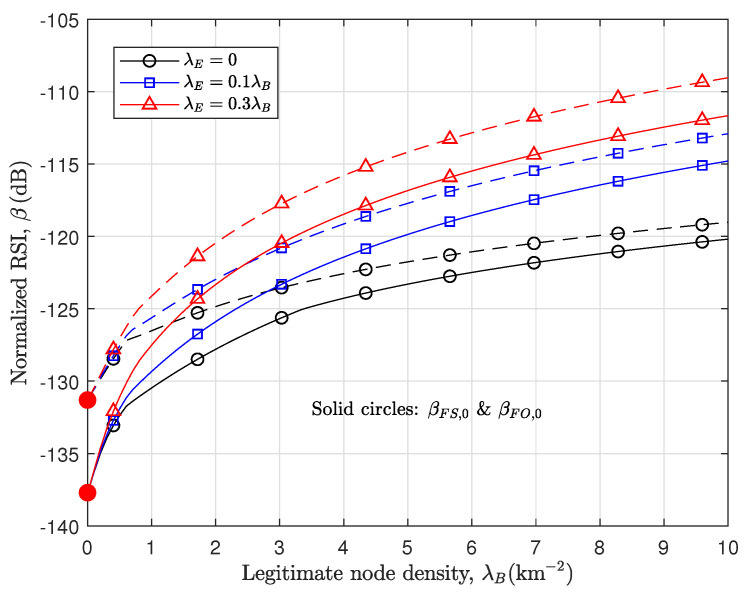
RSI threshold for FD optimality [solid lines] and FD superiority [dashed lines].

Figure 5 examines the impact of $\lambda_{B}$ and $\lambda_{E}$ on the RSI threshold for the FD optimality and superiority. To begin with, the results demonstrate that $\beta_{FO,0}$ and $\beta_{FS,0}$ in Corollaries 3 and 4 provide the exact lower bounds for the FD optimality and superiority condition, each of which guides a reference value for SIC design. In this figure, $\beta_{FO}$ and $\beta_{FS}$ for nonzero interference are numerically obtained through the bisection method, as described in Section 4.2 and Section 5. The results reveal that the condition for $\beta_{FO}$ and $\beta_{FS}$ become looser as the impact of interference increases, and this is consistent with the result in [34]. On the other hand, the results demonstrate that $\beta_{FO}$ and $\beta_{FS}$ in the secure design are significantly affected by the Eve density. As Eves are dense, the FD optimality and superiority conditions become increasingly loose because the RA-WMB tends to work at a higher interference level for confounding Eves. As a result, the contribution of SIC can become further important in the secure RA-WMB.

**Figure 6:**
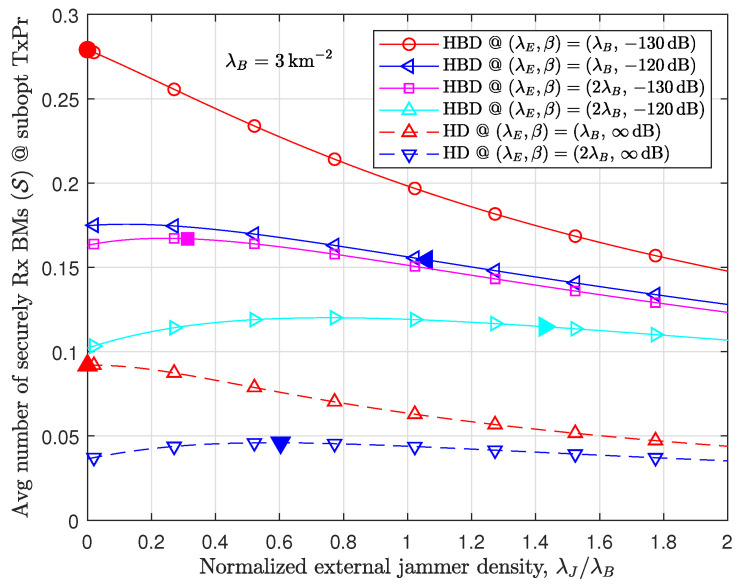
Contribution of external jammers to secure RA-WMB [*y*-axis: S applying ${\overset{˘}{\nu }}_{B}\left(\lambda_{J}\right)$; solid symbols: ${\hat{\lambda }}_{J,F}$ in (23) for HBD and ${\hat{\lambda }}_{J,H}$ derived in [11] for HD].

Figure 6 evaluates the effect of external jammers on increasing S. The value on *y*-axis denotes a suboptimal value of S, which is obtained through applying ${\overset{˘}{\nu }}_{B}$ for a given $\lambda_{J}$ where ${\bar{\nu }}_{\infty }$ in (19) comes from $f_{\bar{\nu },\infty ,J}\left(\nu_{B}\right)=0$ in (A11), and this $\nu_{B}$ is denoted by ${\bar{\nu }}_{\infty }\left(\lambda_{J}\right)$ in this figure; thus, the peak of the curves in Figure 6 represents a suboptimal value for the joint optimization of S over $\nu_{B}$ and $\lambda_{J}$. The results demonstrate that ${\hat{\lambda }}_{J,F}$ designed in Proposition 3 works well in HBD with a low RSI (e.g., β=−130dB) scenario. As RSI increases, ${\hat{\lambda }}_{J,F}$ in (23) deviates from the exact peak, as already expected in Section 4.3. Further, Figure 6 argues that the effect of $\lambda_{J}$ is quite marginal in terms of increasing S, from the observation that S monotonically decreases with $\lambda_{J}$ or the gap between the maximum value and the value for $\lambda_{J}=0$ is not significant, even when the Eve density is large (in this figure, note that $\lambda_{E}=\lambda_{B}$ and $\lambda_{E}=2\lambda_{B}$). This effect is similar to those for HD scenarios, which were obtained from Proposition 3.2 of [11]. As a result, one does not need to consider seriously the deployment of the external jammers for secure RA-WMB because they are marginally helpful when Eves are extremely dense.

## 7. Conclusions

This paper investigated the performance and design principles of the secure random-access-based wireless mutual broadcast (RA-WMB), with the objective of enhancing physical layer security (PLS) performance through self-interference cancellation (SIC). The analysis focused on the impact of SIC on the hybrid duplex (HBD) operation that balances the proportion of legitimate half-duplex (HD) receiving and full-duplex (FD) transceiving nodes. The analytical and numerical results demonstrated that the design of secure HBD RA-WMB differs significantly from conventional designs for nonsecure RA-WMB or secure HD RA-WMB. The deliberate increase in interference in the design, aimed at confounding eavesdroppers, reduced the sensitivity of secrecy performance to residual self-interference. This facilitated a more confident use of SIC, and this paper also examined the condition favoring this FD operation. In contrast, friendly jammers were not helpful in improving PLS performance due to their negative or quite marginal gain, which was similar to the conventional HD-WMB design. Further, this paper proposed a simple method for designing transmission probability, factoring in eavesdropper density and SIC impairment, to enhance the PLS performance. In future work, it would be interesting to expand the study into more interactive scenarios, such as carrier sensing multiple access and Eve collusion, to further generalize scenarios.

## Figures and Tables

**Table 1:** Design principles for secure HBD RA-WMB based on SIC capability.

SIC Capability	Recommended Duplexing	Nonsecure vs. Secure Design	Key Designs
None or bad	Half duplex (HD) ^†^	$\nu_{B,B}^{*}<\nu_{B}^{*}$ ($\nu_{B,B}^{*}\leq \frac{1}{2}$) ^‡^,	$\nu_{B}={\overset{˘}{\nu }}_{B}$ from (19),
(high RSI; e.g., $\beta \gg \beta_{FS}$)	(RxOnly + TxRx with β→∞)	$\nu_{B}^{*}↑\mathrm{as}\;\lambda_{E}↑$	$\beta_{FS}$ from (28) or (30)
Good	Pure hybrid duplex (HBD)	$\nu_{B,B}^{*}<\nu_{B}^{*}$,	$\nu_{B}={\overset{˘}{\nu }}_{B}$ from (19)
(Medium RSI; e.g., $\beta >\beta_{FO}$)	(RxOnly + TxRx)	$\nu_{B}^{*}\;↑\mathrm{as}\;\;\lambda_{E}↑\mathrm{and}\;\beta ↓$	
Excellent	Full duplex (FD)	$\beta_{FO,B}<\beta_{FO}$,	$\beta_{FO}$ from (21) or (22)
(Low RSI; $\beta \leq \beta_{FO}$)	(TxRx, i.e., $\nu_{B}^{*}=1$)	$\beta_{FO}\;↑\mathrm{as}\;\;\lambda_{B}↑\mathrm{and}\;\lambda_{E}↑$	

^†^ The results for HD are equivalent to the results in [11]. ^‡^ Subscript B denotes the one for nonsecure designs.

**Table 2:** Notations and their description for secure HBD RA-WMB.

Notations	Descriptions	Expressions or Values
$S_{o}$	Average number of securely received BMs per node	In (3)
S	Approximation of $S_{o}$ (almost the same as $S_{o}$)	In (6)
$\tilde{S}$, $\hat{S}$	Lower and upper bounds of S	In Proposition 1-(i) and (iii)
B	Average number of nonsecurely received BMs per node	$S(=S_{o})$ for $\lambda_{E}=0$
$S_{H}$, $S_{F}$	S’s for HD and FD	In (5)
H, F, N	RxOnly, TxRx, JamRx modes	-
$\Phi_{B}$, $\Phi_{J}$, $\Phi_{E}$	Sets of legitimate nodes, external jammers, and eavesdroppers	-
$\theta_{d}$	Probability of operation mode d∈{H,F,N}	In (3)
$A_{H}$, $A_{F}$	Area where a receiver can successfully decode BMs if transmitters are located therein, in H and F modes	In (8)
$\lambda_{B}$, $\lambda_{J}$, $\lambda_{E}$	Spatial densities of legitimate nodes, external jammers, Eves	$\lambda_{B}=0.2\;$ to $\;8\;{\mathrm{km}}^{-2}$
*p*, $\delta_{N}p$, $\delta_{J}p$	Tx power of BM, internal jamming, external jamming	p=23dBm, $\delta_{N}=\delta_{J}=1$
α	Path loss exponent	3.5
*K*	Path loss gain at a unit distance 1km	−141.33dBm
${\tilde{\sigma }}^{2}/K$	Normalized noise power ($\sigma^{2}≜\frac{{\tilde{\sigma }}^{2}}{Kp}$)	22.89dBm
$h_{li}$	Rayleigh fading gain from node *i* to node *l*	Unit mean
$\Xi_{B}^{H}$, $\Xi_{B}^{F}$	SINR at legitimate receiver in modes H and F	In (1)
$\Xi_{E}$	SINR at Eve	In (2)
$\xi_{B}$, $\xi_{E}$	SINR thresholds of legitimate receiver and Eve	$\xi_{B}=\xi_{E}=0\;\mathrm{dB}$
β	Residual self-interference (RSI) normalized by *p*	−130 to −100dB
$\beta_{FO}$, $\beta_{FS}$	RSI thresholds for FD optimality and FD superiority	In (22) and (30)
$\beta_{FO,0}$, $\beta_{FS,0}$	$\beta_{FO}$ and $\beta_{FS}$ in a coverage-limited scenario	In (21) and (28)
$\nu_{B}$, $\nu_{N}$	Transmission probability (TxPr), internal jamming probability	$0<\nu_{B}\leq 1$, $\nu_{N}=0$
$\nu_{B}^{*}$, ${\overset{˘}{\nu }}_{B}$	Optimal and suboptimal TxPrs for HBD,	In (17) and (19)
${\bar{\nu }}_{\infty }$	Suboptimal TxPr candidate for HD when $\lambda_{J}=0$ and $\nu_{N}=0$	In Proposition (2)-(ii)

## Data Availability

Data are contained within the article.

## Abbreviations

The following abbreviations are used in this manuscript:

6G	6th generation
AN	Artificial noise
BM	Broadcast message
CSMA	Carrier sense multiple access
D2D	Device-to-device
Eve	Eavesdropper
FD	Full duplex
HBD	Hybrid duplex
HD	Half duplex
HPPP	Homogeneous Poisson point process
IoT	Internet of things
PLS	Physical layer security
pgfl	Probability generating functional
PPP	Poisson point process
RA-WMB	Random access based wireless mutual broadcast
RSI	Residual self-interference
SIC	Self-interference cancellation
TxPr	Transmission probability
V2V	Vehicle-to-vehicle
WMB	Wireless mutual broadcast

## Appendix A. Proof of Lemma 1

In (4), from Campbell’s theorem [27], i.e., $E\left[\sum_{x\in \Phi }f\left(x\right)\right]=\int_{R^{d}}f\left(x\right)\Lambda \left(dx\right)$,

(A1) $$\begin{array}{c} \begin{array}{c} E\left[\sum_{X_{b}\in \Phi_{B}}I\left[d_{b}=F\right]P\left[\Xi_{B}^{d}\left(X_{i}\right)>\xi_{B}\right]\right]=\lambda_{B}\nu_{B}A_{d}\left(\bar{\lambda },\xi_{B}\right). \end{array} \end{array}$$

Furthermore, $A_{d}\left(\bar{\lambda },\xi_{B}\right)$ can be derived as follows:

(A2) $$\begin{array}{c} \begin{array}{cc} A_{d}\left(\bar{\lambda },\xi \right) & ≜\int_{R^{2}}P\left[\Xi_{B}^{d}\left(x\right)>\xi \right]dx\\ \; & =2\pi \int_{0}^{\infty }E\left[\exp\left(-\xi r^{\alpha }\left(I_{B}+I_{J}+\beta_{d}/K+\sigma^{2}\right)\right)\right]rdr\\ \; & \overset{\left(a\right)}{=}2\pi \int_{0}^{\infty }\exp\left(-\xi \left(\beta_{d}/K+\sigma^{2}\right)r^{\alpha }\right)E\left[\prod_{X_{b}\in \Phi_{B}^{\nu_{B}}}\exp\left(-\xi r^{\alpha }h_{ob}{\left|X_{b}\right|}^{-\alpha }\right)\right.\\ \; & \;\left.\cdot \prod_{X_{n}\in \Phi_{B}^{\nu_{N}}}\exp\left(-\xi r^{\alpha }h_{on}{\left|X_{n}\right|}^{-\alpha }\delta_{N}\right)\prod_{X_{j}\in \Phi_{J}}\exp\left(-\xi r^{\alpha }h_{oj}{\left|X_{j}\right|}^{-\alpha }\delta_{J}\right)rdr\right]\\ \; & =2\pi \int_{0}^{\infty }\exp\left(-\xi \left(\beta_{d}/K+\sigma^{2}\right)r^{\alpha }\right)E\left[\prod_{X_{b}\in \Phi_{B}^{\nu_{B}}}\frac{1}{1+\xi r^{\alpha }{\left|X_{b}\right|}^{-\alpha }}\cdot \right.\\ \; & \;\left.\prod_{X_{n}\in \Phi_{B}^{\nu_{N}}}\frac{1}{1+\xi r^{\alpha }{\left|X_{n}\right|}^{-\alpha }\delta_{N}}\prod_{X_{j}\in \Phi_{J}}\frac{1}{1+\xi r^{\alpha }{\left|X_{j}\right|}^{-\alpha }\delta_{J}}\right]rdr\\ \; & \overset{\left(b\right)}{=}2\pi \int_{0}^{\infty }\exp\left(-\pi \bar{\lambda }\xi^{\frac{2}{\alpha }}\Delta \left(\alpha \right)r^{2}-\xi \left(\beta_{d}/K+\sigma^{2}\right)r^{\alpha }\right)rdr\\ \; & \overset{\left(c\right)}{=}\pi \int_{0}^{\infty }\exp\left(-\pi \bar{\lambda }\xi^{\frac{2}{\alpha }}\Delta \left(\alpha \right)x-\xi \left(\beta_{d}/K+\sigma^{2}\right)x^{\frac{\alpha }{2}}\right)dx, \end{array} \end{array}$$

where $\Phi_{s}^{\nu }$ denotes a resultant HPPP with density $\lambda_{s}\nu$ after applying an independent thinning with probability ν to HPPP $\Phi_{s}$, (a) follows from the definition of $I_{B}$ and $I_{J}$ in (1) and the Slivnyak’s theorem [27], (b) follows from the probability generating functional (pgfl) of a PPP, i.e., $E\left[\prod_{x\in \Phi }v\left(x\right)\right]=\exp\left(-\int_{R^{d}}\left(1-v(x)\right)\Lambda \left(dx\right)\right)$, similar to [27,28], and (c) follows from the change of variable, i.e., $x≜r^{2}$.

On the other hand, $E\left[\prod_{Y_{e}\in \Phi_{E}}P\left[\Xi_{E}\left(X_{b},Y_{e}\right)\leq \xi_{E}\right]\right]$ can be obtained as follows:

(A3) $$\begin{array}{c} \begin{array}{cc} \; & E\left[\prod_{Y_{e}\in \Phi_{E}}P\left[\Xi_{E}\left(X_{b},Y_{e}\right)\leq \xi_{E}\right]\right]\overset{\left(a\right)}{=}\exp\left(-\int_{R^{2}}P\left[\Xi_{E}\left(x\right)>\xi_{E}\right]\Lambda \left(dx\right)\right)\\ \; & =\exp\left(-\lambda_{E}\int_{0}^{\infty }P\left[\Xi_{E}\left(x\right)>\xi_{E}\right]dx\right)=\exp\left(-\lambda_{E}A_{H}\left(\bar{\lambda },\xi_{E}\right)\right). \end{array} \end{array}$$

where $\Xi_{E}\left(x\right)$ denotes $\Xi_{E}\left(x,Y\right)$ for Eve *Y* located at the origin, and (a) follows from the fact that the translation of a PPP still constitutes a PPP equivalent to the original one and from the pgfl of a PPP.

As a result, from (A1), (A2), and (A3), (6) and (7) can be obtained.

## Appendix B. Proof of Proposition 1

(i) $s_{d}\left(\nu_{B},\lambda_{N},\nu_{J}\right)>{\overset{ˇ}{s}}_{d}\left(\nu_{B},\lambda_{N},\nu_{J}\right)$ follows from
  (A4) $$\begin{array}{c} \begin{array}{cc} \exp\left(-\lambda_{E}A_{H}\left(\bar{\lambda },\xi_{E}\right)\right)\; & \overset{\left(a\right)}{>}\exp\left(-\pi \lambda_{E}\int_{0}^{\infty }\exp\left(-\pi \bar{\lambda }\xi_{E}^{\frac{2}{\alpha }}\Delta \left(\alpha \right)x\right)dx\right)\\ \; & =\exp\left(-\frac{\lambda_{E}}{\bar{\lambda }\xi_{E}^{\frac{2}{\alpha }}\Delta \left(\alpha \right)}\right)=\exp\left(-\lambda_{E}{\hat{A}}_{H}\left(\bar{\lambda },\xi_{E}\right)\right), \end{array} \end{array}$$
  where (a) follows from $\sigma^{2}>0$.
  Furthermore, ${\overset{ˇ}{s}}_{d}\left(\nu_{B},\lambda_{N},\nu_{J}\right)>{\tilde{s}}_{d}\left(\nu_{B},\lambda_{N},\nu_{J}\right)$ follows from
  (A5) $$\begin{array}{c} \begin{array}{cc} A_{d}\left(\lambda ,\xi \right)\; & =\pi \int_{0}^{\infty }\exp\left(-\pi \lambda \xi^{\frac{2}{\alpha }}\Delta \left(\alpha \right)x-\xi \left(\beta_{d}/K+\sigma^{2}\right)x^{\frac{\alpha }{2}}\right)dx\\ \; & \overset{\left(a\right)}{=}\frac{1}{\lambda \xi^{\frac{2}{\alpha }}\Delta \left(\alpha \right)}E\left[\exp\left(-\xi \left(\beta_{d}/K+\sigma^{2}\right)U^{\frac{\alpha }{2}}\right)\right]\\ \; & \overset{\left(b\right)}{>}\frac{1}{\lambda \xi^{\frac{2}{\alpha }}\Delta \left(\alpha \right)}\exp\left(-E\left[\xi \left(\beta_{d}/K+\sigma^{2}\right)U^{\frac{\alpha }{2}}\right]\right)\\ \; & \overset{\left(c\right)}{=}\frac{1}{\lambda \xi^{\frac{2}{\alpha }}\Delta \left(\alpha \right)}\exp\left(-{\left(\frac{1}{\pi \lambda \Delta (\alpha )}\right)}^{\frac{\alpha }{2}}\Gamma \left(1+\frac{\alpha }{2}\right)\left(\beta_{d}/K+\sigma^{2}\right)\right), \end{array} \end{array}$$
  where (a) follows from the definition of random variable *U* that is exponentially distributed with the mean of $\mu ≜\frac{1}{\pi \lambda \xi^{\frac{2}{\alpha }}\Delta \left(\alpha \right)}$, (b) follows from the Jensen’s inequality, and (c) follows from $E\left[cU^{\frac{\alpha }{2}}\right]=\int_{0}^{\infty }\frac{c}{\mu }u^{\frac{\alpha }{2}}\exp\left(-\frac{u}{\mu }\right)du=c\mu^{\frac{\alpha }{2}}\Gamma \left(1+\frac{\alpha }{2}\right)$.
(ii) If β=0, as $\sigma^{2}\to 0$, the lower bounds in (A4) and (A5) approach the original value.
  Further, when $\nu_{N}$ and $\lambda_{J}$ are fixed, from (12), $\lim_{\sigma^{2}\to 0}S$ for β=0 increases with $\nu_{B}$; thus, $\nu_{B}=1-\nu_{N}$ maximizes $\lim_{\sigma^{2}\to 0}S$ for a given $\nu_{N}$ and $\lambda_{J}$, when β=0.
(iii) This proof is similar to the one for Lemma 3.1(ii) of [11] which is only for HD. However, the result and proof in this paper are for a more general case, i.e., HBD. The inequalities in (13) are indexed as follows:
  (A6) $$\begin{array}{c} \begin{array}{cc} S & \overset{\left(a\right)}{\leq }\lambda_{B}\nu_{B}A_{H}\left(\bar{\lambda },\xi_{B}\right)\exp\left(-\lambda_{E}A_{H}\left(\bar{\lambda },\xi_{E}\right)\right)\\ \; & \overset{\left(b\right)}{\leq }\lambda_{B}\nu_{B}A_{H}\left(\bar{\lambda },\xi_{B}\right)\exp\left(-\lambda_{E}A_{H}\left(\bar{\lambda },\xi_{B}\right)\right)\overset{\left(c\right)}{\leq }\hat{S}. \end{array} \end{array}$$
  Note that $A_{H}\geq \max\left\{A_{F},A_{N}\right\}$ from (8) and β≥0; thus, (a) holds. Further, from the assumption of $\xi_{E}\leq \xi_{B}$, (b) holds. For the proof of (c), consider $f\left(A\right)≜A\exp\left(-\lambda_{E}A\right)$. Because $\frac{\partial f}{\partial A}=\left(1-\lambda_{E}A\right)\exp\left(-\lambda_{E}A\right)$, $\frac{\partial f}{\partial A}>0$ if $A<\frac{1}{\lambda_{E}}$, while $\frac{\partial f}{\partial A}<0$ if $A>\frac{1}{\lambda_{E}}$; thus, the maximum value of f(A) over A is $\frac{1}{\lambda_{E}\exp\left(1\right)}$ which is achieved when $A=\frac{1}{\lambda_{E}}$. Furthermore, this upperbound, i.e., $\frac{\lambda_{B}\nu_{B}}{\lambda_{E}\exp\left(1\right)}$, is maximized at $\nu_{B}=1$. As a result, (iii) holds.
(iv) In (6), a scenario with no interference can be expressed through setting $\bar{\lambda }$ to 0; thus, from (8) for $\bar{\lambda }=0$, (15) is obtained.
  If $\nu_{N}=0$ and $\lambda_{J}=0$,
  (A7) $$\begin{array}{c} \begin{array}{c} S\left(\nu_{B},0,0\right)=\lambda_{B}\nu_{B}\left(\left(1-\nu_{B}\right)A_{H}\left(0,\xi_{B}\right)+\nu_{B}A_{F}\left(0,\xi_{B}\right)\right)\exp\left(-\lambda_{E}A_{H}\left(0,\xi_{E}\right)\right). \end{array} \end{array}$$
  When considering that both $A_{H}\left(0,\xi \right)$ and $A_{F}\left(0,\xi \right)$ are now independent of $\nu_{B}$, $S(\nu_{B},0,0)$ is log-concave for $0<\nu_{B}\leq 1$. As a result, $S(\nu_{B},0,0)$ can be maximized at $\nu_{B}=1$ or such $\nu_{B}$ that
  (A8) $$\begin{array}{c} \begin{array}{c} \frac{\partial }{\partial \nu_{B}}\log S\left(\nu_{B},0,0\right)=\frac{1}{\nu_{B}}+\frac{A_{H}\left(0,\xi_{B}\right)-A_{F}\left(0,\xi_{B}\right)}{\left(A_{H}\left(0,\xi_{B}\right)-A_{F}\left(0,\xi_{B}\right)\right)\nu_{B}-A_{H}\left(0,\xi_{B}\right)}=0. \end{array} \end{array}$$
  From (A8), (16) is derived.
(v) Consider that $S\left(1,0,0\right)=\lambda_{B}A_{F}\left(\lambda_{B},\xi_{B}\right)\exp\left(-\lambda_{E}A_{H}\left(\lambda_{B},\xi_{E}\right)\right)$ and $S_{H}\left(\nu_{B},0,0\right)=\lambda_{B}\nu_{B}\left(1-\nu_{B}\right)A_{H}\left(\lambda_{B}\nu_{B},\xi_{B}\right)\exp\left(-\lambda_{E}A_{H}\left(\lambda_{B}\nu_{B},\xi_{E}\right)\right)$.
  From Propositons 1(i) and 1(iii) of [34], when β=0, B for FD always outperforms the maximum B for HD, i.e., $\lambda_{B}A_{F}\left(\lambda_{B},\xi_{B}\right)>\lambda_{B}\nu_{B}\left(1-\nu_{B}\right)A_{H}\left(\lambda_{B}\nu_{B},\xi_{B}\right)$ for any $\nu_{B}$. Further, $\exp\left(-\lambda_{E}A_{H}\left(\lambda_{B},\xi_{E}\right)\right)>\exp\left(-\lambda_{E}A_{H}\left(\lambda_{B}\nu_{B},\xi_{E}\right)\right)$ for $0<\nu_{B}<1$ because $A_{H}$ decreases as transmitter density increases. Therefore, when β=0, $S\left(1,0,0\right)\geq \max_{\nu_{B}}S_{H}\left(\nu_{B},0,0\right)$.
  Furthermore, S(1,0,0) is continuous and monotonically decreases with respect to β, and $\lim_{\beta \to \infty }S\left(1,0,0\right)=0$.
  As a result, Proposition 1-(v) holds.

## Appendix C. Proof of Proposition 2

Proposition 2 assumes that $\lambda_{J}=0$, but this proof assumes that $\lambda_{J}\geq 0$ is given for a further general result. In particular, these results are also used for obtaining some numerical results in Section 6.

When $\bar{\nu }≜\nu_{B}+\frac{\lambda_{J}}{\lambda_{B}}\delta_{J}^{\frac{2}{\alpha }}$, $\bar{\lambda }=\lambda_{B}\bar{\nu }$ and $\frac{\partial \bar{\nu }}{\partial \nu_{B}}=1$. Then,

(A9) $$\begin{array}{c} \begin{array}{cc} \log\tilde{S}\left(\nu_{B},0,\lambda_{J}\right) & =\Theta +\log\left(\frac{\nu_{B}}{\bar{\nu }}\right)-\bar{\Omega }\sigma^{2}{\bar{\nu }}^{-\frac{\alpha }{2}}-\Lambda_{E}{\bar{\nu }}^{-1}\\ \; & \;+\log\left(\left(1-\nu_{B}\right)+\nu_{B}\exp\left(-\frac{\bar{\Omega }\beta }{K}{\bar{\nu }}^{-\frac{\alpha }{2}}\right)\right). \end{array} \end{array}$$

where $\bar{\Omega }≜\frac{2}{\alpha }\Omega$. The partial derivative of $\log\tilde{S}$ with respect to $\nu_{B}$ is given as

(A10) $$\begin{array}{c} \begin{array}{cc} \; & f_{{\tilde{\nu }}_{B}}\left(\nu_{B}\right)≜\frac{\partial \log\tilde{S}\left(\nu_{B},0,\lambda_{J}\right)}{\partial \nu_{B}}\\ \; & =\nu_{B}^{-1}-{\bar{\nu }}^{-1}+\Omega \sigma^{2}{\bar{\nu }}^{-\frac{\alpha }{2}-1}+\Lambda_{E}{\bar{\nu }}^{-2}+\frac{-1+\left(1+\frac{\Omega \beta }{K}\nu_{B}{\bar{\nu }}^{-\frac{\alpha }{2}-1}\right)\exp\left(-\frac{\bar{\Omega }\beta }{K}{\bar{\nu }}^{-\frac{\alpha }{2}}\right)}{\left(1-\nu_{B}\right)+\nu_{B}\exp\left(-\frac{\bar{\Omega }\beta }{K}{\bar{\nu }}^{-\frac{\alpha }{2}}\right)}\\ \; & =\nu_{B}^{-1}-{\bar{\nu }}^{-1}+\Omega \sigma^{2}{\bar{\nu }}^{-\frac{\alpha }{2}-1}+\Lambda_{E}{\bar{\nu }}^{-2}+\frac{1+\frac{\Omega \beta }{K}\nu_{B}{\bar{\nu }}^{-\frac{\alpha }{2}-1}-\exp\left(\frac{\bar{\Omega }\beta }{K}{\bar{\nu }}^{-\frac{\alpha }{2}}\right)}{\nu_{B}+\left(1-\nu_{B}\right)\exp\left(\frac{\bar{\Omega }\beta }{K}{\bar{\nu }}^{-\frac{\alpha }{2}}\right)}. \end{array} \end{array}$$

(i) From (A10), when β=0, $\frac{\partial \log\tilde{S}\left(\nu_{B},0,\lambda_{J}\right)}{\partial \nu_{B}}=\nu_{B}^{-1}-{\bar{\nu }}^{-1}+\Omega \sigma^{2}{\bar{\nu }}^{-\frac{\alpha }{2}-1}+\Lambda_{E}{\bar{\nu }}^{-2}>0$ for all $\nu_{B}$ because $\nu_{B}^{-1}\geq {\bar{\nu }}^{-1}$. Further, note that $\frac{\partial \log\tilde{S}\left(\nu_{B},0,\lambda_{J}\right)}{\partial \nu_{B}}$ is continuous with respect to β; thus, there exists an $\beta_{c,J}>0$ such that it always holds that $\frac{\partial \log\tilde{S}\left(\nu_{B},0,\lambda_{J}\right)}{\partial \nu_{B}}>0$ if $0\leq \beta \leq \beta_{c,J}$.
(ii) This is the same as the result in Proposition 3.1 of [11]. The proof is fully provided due to its completeness and differences in notation. Assume that β→∞. Then, each term of $\log\tilde{S}\left(\nu_{B},0,\lambda_{J}\right)$ in (A9) is concave for $0<\nu_{B}<1$; thus, $\tilde{S}\left(\nu_{B},0,0\right)$ is log-concave for $0<\nu_{B}<1$, and if the solution of $\frac{\partial \log\tilde{S}\left(\nu_{B},0,\lambda_{J}\right)}{\partial \nu_{B}}=0$ exists between 0 and 1, it is unique. Note that $\frac{\partial \log\tilde{S}\left(\nu_{B},0,\lambda_{J}\right)}{\partial \nu_{B}}=0$ can be recast into $f_{\bar{\nu },\infty ,J}\left(x\right)=0$, where
  (A11) $$\begin{array}{c} \begin{array}{cc} \;f_{\bar{\nu },\infty ,J}\left(\nu_{B}\right) & ≜\left(2\nu_{B}-1\right){\bar{\nu }}^{\frac{\alpha }{2}+1}+\nu_{B}\left(1-\nu_{B}\right){\bar{\nu }}^{\frac{\alpha }{2}}-\nu_{B}\left(1-\nu_{B}\right)\Lambda_{E}{\bar{\nu }}^{\frac{\alpha }{2}-1}-\nu_{B}\left(1-\nu_{B}\right)\Omega \sigma^{2}. \end{array} \end{array}$$
  Similarly, if there exists a $\nu_{B}$ such that $f_{\bar{\nu },\infty ,J}\left(\nu_{B}\right)=0$ for $0<\nu_{B}<1$, it is unique. When $\lambda_{J}=0$, $f_{\bar{\nu },\infty ,J}\left(\nu_{B}\right)=0$ is equivalent to $f_{\bar{\nu },\infty }\left(\nu_{B}\right)=0$. As a result, if $\lambda_{J}=0$, $\tilde{S}$ can be maximized through setting $\nu_{B}$ to ${\bar{\nu }}_{\infty }$ or one.
(iii) ${\tilde{\nu }}_{B}\in {\tilde{N}}_{\nu_{B}}$ is a necessary condition for maximizing S.

## Appendix D. Proof of Proposition 3

Assume that $\nu_{B}$ is fixed. Let $\bar{\nu }≜\nu_{B}+\frac{\lambda_{J}}{\lambda_{B}}\delta_{J}^{\frac{2}{\alpha }}$, $x≜\frac{1}{\bar{\nu }}$, and $\bar{\Omega }≜\frac{2}{\alpha }\Omega$. Then, $0<x\leq \frac{1}{\nu_{B}}$, and from (A9),

(A12) $$\begin{array}{c} \begin{array}{c} \log\tilde{S}\left(\nu_{B},0,\lambda_{J}\right)=\Theta +\log\left(\nu_{B}x\right)-\bar{\Omega }\sigma^{2}x^{\frac{\alpha }{2}}-\Lambda_{E}x+\log\left(\left(1-\nu_{B}\right)+\nu_{B}\exp\left(-\bar{\Omega }\frac{\beta }{K}x^{\frac{\alpha }{2}}\right)\right) \end{array} \end{array}$$

which is log-concave for 0<x≤1 if $\nu_{B}=1$. Furthermore,

(A13) $$\begin{array}{c} \begin{array}{c} \frac{\partial \log\tilde{S}\left(\nu_{B},0,\lambda_{J}\right)}{\partial x}=-\Omega \sigma^{2}x^{\frac{\alpha }{2}-1}-\Lambda_{E}+\frac{1}{x}-\frac{\Omega \frac{\beta }{K}\nu_{B}x^{\frac{\alpha }{2}-1}\exp\left(-\bar{\Omega }\frac{\beta }{K}x^{\frac{\alpha }{2}}\right)}{\left(1-\nu_{B}\right)+\nu_{B}\exp\left(-\bar{\Omega }\frac{\beta }{K}x^{\frac{\alpha }{2}}\right)}. \end{array} \end{array}$$

Furthermore, $\frac{\partial \log\tilde{S}\left(\nu_{B},0,\lambda_{J}\right)}{\partial x}=0$ is equivalent to $f_{J,\nu_{B}}\left(x\right)=0$, where

(A14) $$\begin{array}{c} \begin{array}{c} f_{J,\nu_{B}}\left(x\right)≜\Omega \sigma^{2}x^{\frac{\alpha }{2}}+\Lambda_{E}x-1+\frac{\Omega \frac{\beta }{K}\nu_{B}x^{\frac{\alpha }{2}}\exp\left(-\Omega \frac{\beta }{K}x^{\frac{\alpha }{2}}\right)}{\left(1-\nu_{B}\right)+\nu_{B}\exp\left(-\Omega \frac{\beta }{K}x^{\frac{\alpha }{2}}\right)}. \end{array} \end{array}$$

Therefore, $f_{J,\nu_{B}}\left(x\right)$ becomes $f_{J}\left(x\right)$ in (24) when $\nu_{B}=1$. Note that when $\nu_{B}=1$, from the log-concavity of $\tilde{S}\left(\nu_{B},0,\lambda_{J}\right)$ for 0<x≤1, if the solution of $f_{J,\nu_{B}}\left(x\right)=0$ for 0<x≤1 exists, it is unique. Further, it should be met that $f_{J}\left(1\right)\geq 0$ for the existence of $0<\hat{x}\leq 1$ because $f_{J}\left(0\right)=-1$.

As a result, from the relationship between $\lambda_{J}$ and $\hat{x}$ and the condition of $f_{J}\left(1\right)\geq 0$, (23) is obtained.
